# Beyond the Influence of *IDH* Mutations: Exploring Epigenetic Vulnerabilities in Chondrosarcoma

**DOI:** 10.3390/cancers12123589

**Published:** 2020-11-30

**Authors:** Sanne Venneker, Alwine B. Kruisselbrink, Zuzanna Baranski, Ieva Palubeckaite, Inge H. Briaire-de Bruijn, Jan Oosting, Pim J. French, Erik H. J. Danen, Judith V. M. G. Bovée

**Affiliations:** 1 Department of Pathology, Leiden University Medical Center, 2333 ZA Leiden, The Netherlands; S.Venneker@lumc.nl (S.V.); A.B.Kruisselbrink@lumc.nl (A.B.K.); I.Palubeckaite@lumc.nl (I.P.); I.H.Briaire-de_Bruijn@lumc.nl (I.H.B.-d.B.); J.Oosting@lumc.nl (J.O.); 2 Division of Drug Discovery and Safety, Leiden Academic Centre for Drug Research, Leiden University, 2333 CC Leiden, The Netherlands; z.baranski@elsevier.com (Z.B.); e.danen@lacdr.leidenuniv.nl (E.H.J.D.); 3 Department of Neurology, Erasmus University Medical Center, 3015 GD Rotterdam, The Netherlands; p.french@erasmusmc.nl

**Keywords:** sarcoma, enchondroma, chondrosarcoma, isocitrate dehydrogenase, epigenetics, histone deacetylase, B-cell lymphoma-2 family, D-2-hydroxyglutarate, AGI-5198, romidepsin

## Abstract

**Simple Summary:**

Cartilage tumors frequently harbor mutations in the isocitrate dehydrogenase *(IDH1* or *IDH2*) genes. These mutations cause an increase in the levels of the oncometabolite D-2-hydroxyglutarate (D-2-HG), which leads to widespread changes in several cellular processes, including the epigenetic landscape. The aim of our study was to explore whether the methylome of *IDH* mutant cartilage tumors is altered upon tumor progression and whether these underlying epigenetic vulnerabilities could be used as a target for therapy in both *IDH* wildtype and *IDH* mutant high-grade chondrosarcomas. As surgery is nowadays the only treatment option for chondrosarcoma patients, the identification of novel therapeutic strategies remains an important endeavor. The findings in this study show that histone deacetylase (HDAC) inhibition may represent a promising therapeutic strategy for all chondrosarcoma patients, since sensitivity towards this therapeutic option seems independent of the *IDH* mutation status and the chondrosarcoma subtype.

**Abstract:**

Mutations in the isocitrate dehydrogenase (*IDH1* or *IDH2*) genes are common in enchondromas and chondrosarcomas, and lead to elevated levels of the oncometabolite D-2-hydroxyglutarate causing widespread changes in the epigenetic landscape of these tumors. With the use of a DNA methylation array, we explored whether the methylome is altered upon progression from *IDH* mutant enchondroma towards high-grade chondrosarcoma. High-grade tumors show an overall increase in the number of highly methylated genes, indicating that remodeling of the methylome is associated with tumor progression. Therefore, an epigenetics compound screen was performed in five chondrosarcoma cell lines to therapeutically explore these underlying epigenetic vulnerabilities. Chondrosarcomas demonstrated high sensitivity to histone deacetylase (HDAC) inhibition in both 2D and 3D in vitro models, independent of the *IDH* mutation status or the chondrosarcoma subtype. siRNA knockdown and RNA expression data showed that chondrosarcomas rely on the expression of multiple HDACs, especially class I subtypes. Furthermore, class I HDAC inhibition sensitized chondrosarcoma to glutaminolysis and Bcl-2 family member inhibitors, suggesting that HDACs define the metabolic state and apoptotic threshold in chondrosarcoma. Taken together, HDAC inhibition may represent a promising targeted therapeutic strategy for chondrosarcoma patients, either as monotherapy or as part of combination treatment regimens.

## 1. Introduction

Chondrosarcomas are bone malignancies characterized by the production of cartilage and account for 20% of all malignant bone tumors [1,2]. Based on the anatomical location and pathological characteristics of the tumors, chondrosarcomas are divided into several subtypes: conventional chondrosarcoma (85%), dedifferentiated chondrosarcoma (10%), and rare subtypes (5%), which include mesenchymal-, clear cell-, and periosteal chondrosarcomas. Conventional chondrosarcomas are classified into central (85%) and peripheral (15%) tumors, based on their anatomical location (medulla or surface of the bone, respectively) [1,2]. The most important factor to predict metastatic potential and overall survival of patients with conventional chondrosarcoma is histological grading, which is defined by the cellularity and matrix formation of the tumor. Patients with atypical cartilaginous tumor (ACT)/chondrosarcoma grade I have a low metastatic rate and an overall 10-year survival rate of 88–95% [1,3]. However, patients with high-grade tumors (i.e., grade II and grade III) have an increased metastasis rate (10% and 71%, respectively) and a decreased overall 10-year survival rate (58–86% and 26–55%, respectively) [2,3]. This worse prognosis can partially be ascribed to the intrinsic chemo- and radiotherapy resistance of chondrosarcomas and the lack of targeted therapeutic options. Currently, the only curative treatment option for chondrosarcoma patients is surgery [4], which underlines the need to develop novel targeted therapeutic strategies, in particular for patients with unresectable or high-grade tumors.

The most common genetic alteration in central conventional chondrosarcoma is the hotspot mutation affecting the arginine residues encoded by the isocitrate dehydrogenase 1 and -2 (*IDH1* or *IDH2,* collectively referred to as *IDH*) genes (R132 and R140/R172, respectively), occurring in ~50% of the cases [5,6]. The IDH2 enzyme metabolizes isocitrate to α-ketoglutarate (α-KG) and CO_2_ in the tricarboxylic acid cycle, while the IDH1 enzyme is located in the cytosol where it performs a similar reaction. The hotspot mutations induce a gain-of-function of these enzymes, resulting into abnormal cellular concentrations of the oncometabolite D-2-hydroxyglutarate (D-2-HG) [7]. Chondrosarcomas produce relatively high levels of D-2-HG: approximately 40% of the chondrosarcomas harbor an *IDH1*^R132C^ mutation, which is one of the most efficient D-2-HG producers [8,9]. 

Due to the high structural similarity between α-KG and its antagonist D-2-HG, α-KG dependent enzymes are inhibited by the high levels of the oncometabolite [10,11], causing widespread changes in the metabolic state, DNA damage repair mechanisms, and growth signaling pathways [12,13]. The epigenetic landscape of *IDH* mutated cells is highly altered due to this competitive inhibition of α-KG dependent DNA demethylases (family of TET enzymes) and histone demethylases (family of Jumonji enzymes) [10,13], leading to an aberrant methylation pattern. This indicates that *IDH* mutations interfere with the dynamic processes that regulate the accessibility of the DNA and could thereby permanently change gene expression, which could ultimately lead to tumor formation and growth. Indeed, the introduction of an *IDH* mutation induces the formation of benign cartilage tumors (i.e., enchondromas) in mice [14]. *IDH* mutated enchondromas, which are considered as the benign pre-cursor lesions of chondrosarcoma, are characterized by a CpG island methylator phenotype (CIMP)-positive status [6]. The mutation shifts the differentiation of mesenchymal stem cells, the presumed cells-of-origin in cartilage tumors, towards the chondrogenic lineage [15,16]. Together, these findings indicate that the *IDH* mutation is an early genetic event which highly alters the epigenetic landscape in early phases of cartilage tumor formation. The characteristic hypermethylation phenotype is also observed in primary *IDH* mutant chondrosarcomas [17]. Therefore, targeting the epigenetic changes induced by *IDH* mutations might be beneficial for chondrosarcoma patients, and could potentially help to overcome the intrinsic chemotherapy resistance in chondrosarcoma. 

In this study, we confirmed that the hypermethylation phenotype is retained upon progression from *IDH* mutant enchondroma towards chondrosarcoma using a methylation array on primary tumor samples. In fact, high grade tumors demonstrated an increased number of hypermethylated genes as compared to low grade tumors, suggesting that epigenetic mechanisms play an important role in chondrosarcoma progression. Therefore, a broad compound screen containing 128 compounds which target different epigenetic key players (including histone deacetylases (HDACs), sirtuins (SIRTs), histone demethylases (HDMs), histone acetyltransferases (HATs), histone methyltransferases (HMTs), and DNA methyltransferases (DNMTs)) was performed on *IDH* wild type and mutant chondrosarcoma cell lines to explore whether these epigenetic changes can be used as a target for novel anti-cancer therapy. No synthetic lethal interactions with *IDH* mutations were identified, but several general interesting targets were determined, and interesting compound classes included HDAC and bromodomain and extra-terminal motif (BET) protein inhibitors. Additionally, we explored if one of the most promising hits, the HDAC inhibitor romidepsin, could help to sensitize chondrosarcoma cells to chemotherapy or small molecule inhibitors. To address this question, a drug screen with a panel of non-epigenetic drugs was designed to explore if a combination treatment could be a promising therapeutic strategy for chondrosarcoma patients.

## 2. Results

### 2.1. IDH Mutant Chondrosarcomas Have Increased Hypermethylation with Increasing Histological Grade

**Figure 1:**
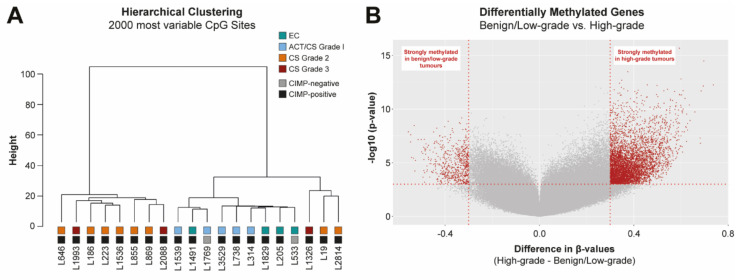
*IDH* mutant chondrosarcomas have increased hypermethylation with increasing histological grade. (**A**) Hierarchical clustering of the methylome of *IDH* mutant primary cartilage tumors (*n* = 20) showed a cluster dominated by benign/low-grade cartilage tumors and a cluster of solely high-grade tumors. Almost all tumors had a CIMP-positive status. Clustering was based on the 2000 most variable CpG sites and performed with the Ward’s method. EC: enchondroma, ACT: atypical cartilaginous tumor, CS: chondrosarcoma. (**B**) Volcano plot of differentially methylated genes between benign/low-grade and high-grade cartilage tumors. Vertical red lines indicate a difference in β-values of at least 0.3 between the two groups. Significantly differentially methylated genes are indicated with red (cut-off at *p* < 0.001). A gene list can be found in Table S2.

CIMP-status analysis shows that almost all *IDH* mutant cartilage tumors were CIMP-positive, except for one enchondroma and one low-grade chondrosarcoma (L533 and L1769, respectively) (Figure 1A, Table S1). Hierarchical clustering of the methylome of these 20 *IDH* mutant primary cartilage tumors resulted in two clusters: a group dominated by benign/low-grade cartilage tumors and a group consisting of solely high-grade chondrosarcomas (Figure 1A). Thus, even though all tumors contained a mutation in *IDH*, and most of them were CIMP-positive, the methylation status was strongly influenced by the histological grade. When comparing the two groups, it turned out that high-grade tumors were more strongly methylated for 592 genes, while they were less strongly methylated for 89 genes (Figure 1B; gene lists are shown in Table S2). Of note, grade III chondrosarcomas were even more strongly methylated than grade II chondrosarcomas (Figure S1A). Biological pathway analysis showed that especially signal transduction and inflammation related genes were affected by increased promoter methylation (Figure S1B). Hence, epigenetic mechanisms seem to play an important role in chondrosarcoma progression. To further explore these underlying epigenetic vulnerabilities, we took a screening-based approach to identify epigenetic regulators that play a role in high-grade chondrosarcoma.

### 2.2. Epigenetic Compound Screening Identifies HDAC Enzymes as Important Epigenetic Regulators in Chondrosarcoma, Independent of the IDH Mutation Status 

**Figure 2:**
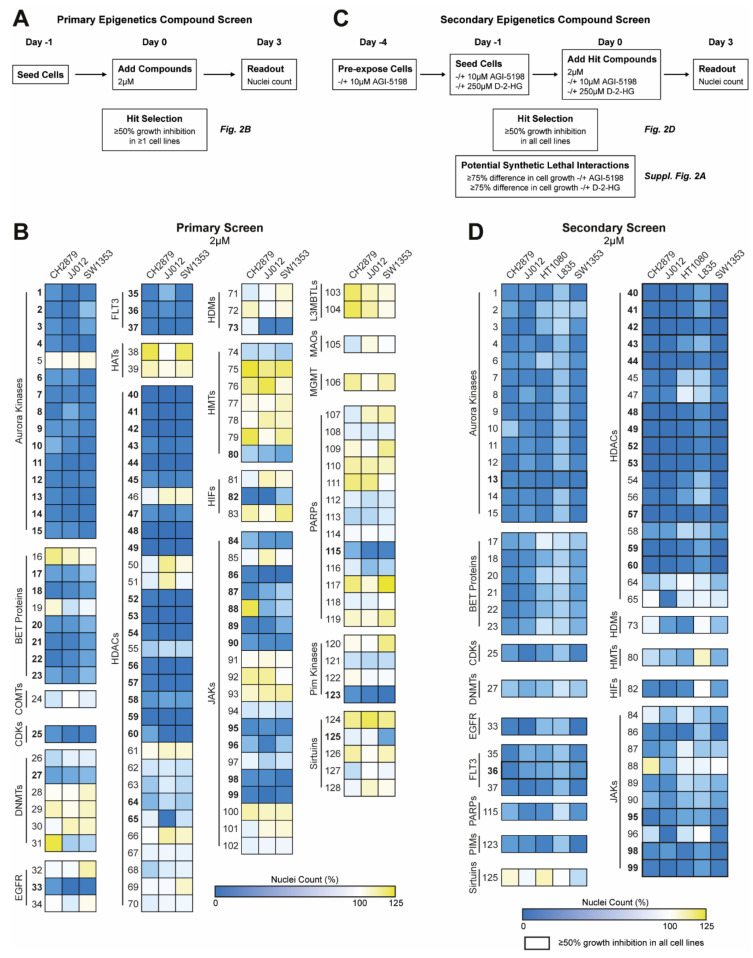
Drug screening identifies HDAC enzymes as important epigenetic regulators in chondrosarcoma, independent of the *IDH* mutation status. (**A**) Schematic overview of the performed primary epigenetics compound screen. (**B**) Heatmaps of the results from the primary epigenetics compound screen, in which blue indicates inhibition of cell growth and yellow an induction of cell growth. Three chondrosarcoma cell lines were treated with 128 compounds at a concentration of 2 µM for 72 h. Aurora kinase, BET protein, FLT3, HDAC, and JAK inhibitors were identified as interesting compound classes. All compounds (*n* = 61) that inhibited growth ≥50%, indicated by the bold numbers, were selected for the secondary screen. (**C**) Schematic overview of the performed secondary epigenetics compound screen. (**D**) Heatmaps of the results from the secondary epigenetics compound screen. Five chondrosarcoma cell lines were treated with 61 compounds at a concentration of 2 µM for 72 h. All compounds that reduced the nuclei count ≥50% in all cell lines were identified as hit (*n* = 17). HDAC inhibitors were identified as the most promising compound class (*n* = 12).

The primary epigenetics compound screen was performed as described in Figure 2A. The results show that several compound classes reduced the growth of all chondrosarcoma cell lines, including Aurora kinase inhibitors, BET protein inhibitors, Fms Related Receptor Tyrosine Kinase 3 (FLT3) inhibitors, HDAC inhibitors, and Janus kinase (JAK) inhibitors (Figure 2B). Of note, DNA methyltransferase (DNMT) inhibitors did not have a pronounced effect on chondrosarcoma cell growth, except for #27 (decitabine). All compounds that inhibited growth with ≥50%, as indicated by the bold numbers in Figure 2B, were selected for the secondary epigenetics compound screen (Figure 2C). 

The secondary screen identified 17 compounds that reduced cell growth with ≥50% in all five chondrosarcoma cell lines, among which the HDAC inhibitors represented the biggest group (Figure 2D). Furthermore, the secondary screen showed that the effect of the compounds seemed independent of the *IDH* mutation status, as only one potential synthetic lethal interaction was observed in JJ012 cells (#65, tubacin) (Figure S1A). However, dose–response curves of tubacin in IDH1 mutant cell lines treated with or without the IDH1 mutant inhibitor AGI-5198 [18] did not confirm the rescue observed in the secondary epigenetics compounds screen (Figure S2B). Hence, these results suggest that targeting of epigenetic regulators in chondrosarcoma, especially the HDAC enzymes, could be a promising therapeutic strategy, irrespective of the *IDH* mutation status. 

### 2.3. Chondrosarcoma Cell Lines Mainly Rely on the Expression of HDAC Class I Subtypes 

**Figure 3:**
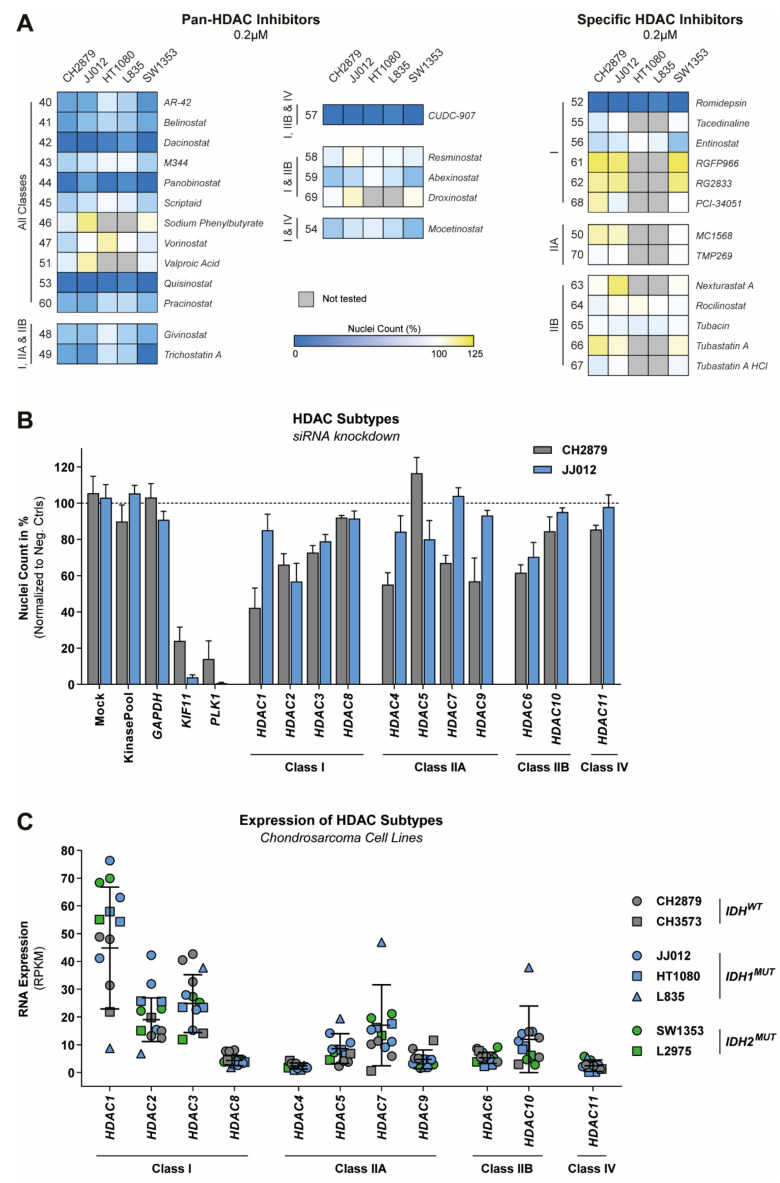
Chondrosarcoma cell lines mainly rely on the expression of HDAC Class I subtypes. (**A**) Heatmap that visualizes the results of five chondrosarcoma cell lines which were treated with all HDAC inhibitors included in epigenetics compound screen (*n* = 31) at a concentration of 0.2 µM for 72 h. Blue indicates inhibition of cell growth and yellow an induction of cell growth. Grey indicates that an inhibitor was not tested in that specific cell line. Inhibitors were sorted on the specific HDAC classes that are targeted by these compounds. Chondrosarcoma cell lines are sensitive to several pan-HDAC inhibitors and the selective class I HDAC inhibitor romidepsin. (**B**) Knockdown of all single HDAC subtypes with siRNA SMARTpools and DharmaFECT 3 in two chondrosarcoma cell lines did not have a prominent effect on chondrosarcoma cell line growth. Mock, siGAPDH, and siKinasePool were used as negative controls, and siKIF11 and siPLK1 were used as positive controls. Bars represent the mean of one experiment performed in triplicate ± standard deviation. (**C**) RNA expression of all HDAC subtypes per cell line determined from a previously published RNA sequencing data set [19]. Class I HDAC subtypes were most abundantly expressed in chondrosarcoma cell lines.

The human genome encodes for eleven subtypes of HDAC enzymes, which can be divided into four different classes: class I (HDAC1, -2, -3, and -8), class IIA (HDAC4, -5, -7, and -9), class IIB (HDAC6 and HDAC10), and class IV (HDAC11). To identify the most potent and specific HDAC inhibitor for follow-up studies, all HDAC inhibitors were screened at a lower concentration (i.e., 0.2 µM) and ordered based on HDAC class specificity (Table S3). As shown in Figure 3A, chondrosarcoma cell lines were sensitive to HDAC inhibitors that target multiple classes. Interestingly, one specific class I HDAC inhibitor (i.e., romidepsin) reached a similar level of growth inhibition in all cell lines as some pan-HDAC inhibitors. To evaluate if one of the eleven HDAC subtypes plays the most pivotal role in chondrosarcoma cell survival, siRNA knockdown for each subtype was performed in CH2879 and JJ012 cell lines. Knockdown of a single HDAC subtype did not have a prominent effect on chondrosarcoma cell line growth (Figure 3B), which suggests that chondrosarcoma cell lines rely on multiple HDAC subtypes to maintain cellular growth. Of note, knockdown of the class I subtypes HDAC1 and HDAC2 had the most pronounced effect on the growth of CH2879 cells and JJ012 cells, respectively (Figure 3B). Additionally, analysis of previously published RNA sequencing data [19] showed that chondrosarcoma cell lines expressed all 11 HDAC subtypes, among which class I was most abundantly expressed (Figure 3C). Overall, this data indicates that multiple HDAC subtypes play a pivotal role in chondrosarcoma cell growth and highlights class I as the most important subgroup. Therefore, the class I HDAC inhibitor romidepsin was selected for further validation studies.

### 2.4. Chondrosarcoma Cell Lines Are Sensitive to Romidepsin, Irrespective of the Chondrosarcoma Subtype or IDH Mutation Status

**Figure 4:**
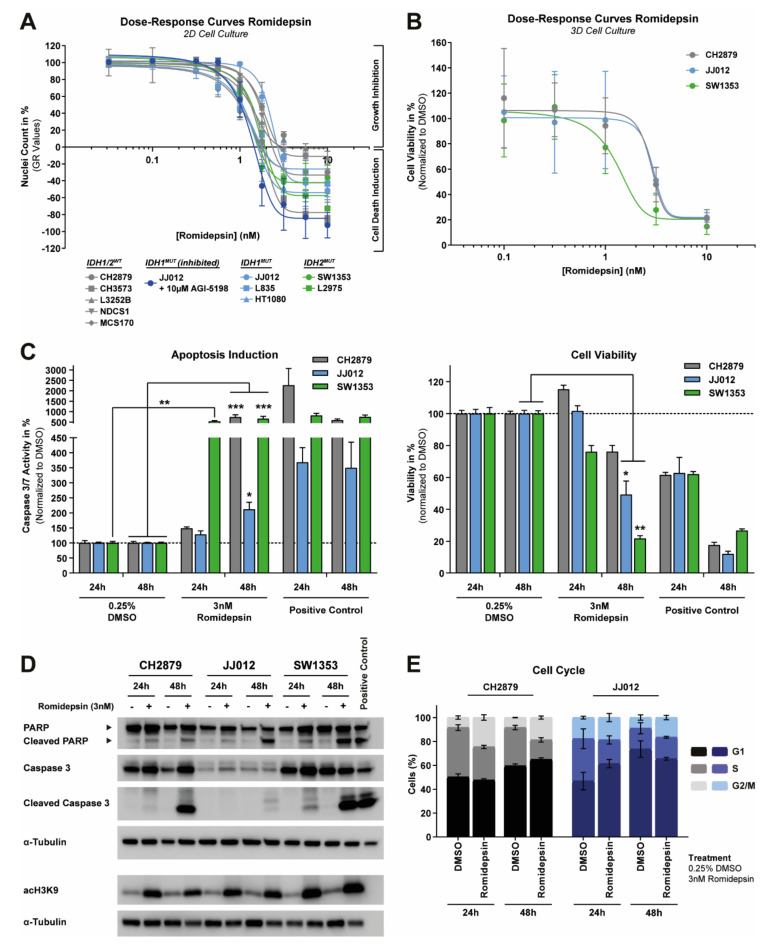
Romidepsin inhibits growth, induces apoptosis and causes a cell cycle arrest in chondrosarcoma cell lines. (**A**) Dose–response curves of romidepsin after 72 h of treatment for 10 chondrosarcoma cell lines cultured in 2D. Chondrosarcoma cell lines were highly sensitive to romidepsin treatment, and the effect could not be rescued if the JJ012 cell line was treated long-term with 10 µM AGI-5198 (>20 passages). Data were corrected for growth rate (GR values) and GR_50_ values were calculated. Data points represent the mean of three experiments performed in triplicate ± standard deviation. (**B**) Dose–response curves of romidepsin after 72 h of treatment for three chondrosarcoma cell lines cultured in 3D. Romidepsin sensitivity was comparable between 2D and 3D culture conditions. Data points represent the mean of four experiments performed in triplicate ± standard deviation. (**C**) Cleaved caspase 3/7 activity and corresponding viability after 24 h and 48 h treatment with 3 nM romidepsin. All cell lines showed a significant increase in apoptosis after 48 h of treatment. As a positive control, treatment with 5µM ABT-737 + 1µM doxorubicin was used. Bars represent the mean of three experiments performed in duplicate ± standard deviation. Significant changes were determined with a Kruskal–Wallis/Dunn’s test: * = *p* < 0.05, ** = *p* < 0.01, *** = *p* < 0.001, **** = *p* < 0.0001. (**D**) Western blot for full-length/cleaved PARP, full-length/cleaved caspase 3 and acH3K9 after 24 h and 48 h treatment with 3 nM romidepsin. All cell lines showed an induction in histone H3 acetylation and apoptosis after 24 h or 48 h of treatment, respectively. As a positive control for apoptosis induction, CH2879 cells treated for 24 h with 5µM ABT-737 + 1µM doxorubicin were used. α-Tubulin was used as a loading control. Whole blots with densitometry readings can be found in Figure S7A. (**E**) Cell cycle analysis after 3 nM romidepsin treatment for 24 h or 48 h. Romidepsin caused a G1 or G2/M phase cell cycle arrest, depending on the time-point and cell line. Bars represent the mean of three independent experiments ± standard deviation.

Romidepsin is clinically approved for the treatment of cutaneous T-cell lymphoma and other peripheral T-cell lymphomas, and is highly selective for binding class I subtypes as compared to the other HDAC classes [20,21]. Dose–response curves showed that all chondrosarcoma cell lines were highly sensitive to romidepsin with GR_50_ values in the range of 0.89 to 1.96 nM after 72 h of treatment (Figure 4A and Table 1). The response to romidepsin treatment was independent of chondrosarcoma subtype and the *IDH* mutation status, as the effect could not be rescued if JJ012 was treated long-term with AGI-5198 (Figure 4A and Table 1). Furthermore, dose–response curves of romidepsin in 3D cell cultures of CH2879, JJ012, and SW1353 showed similar IC_50_ values as compared to the values obtained in the 2D cell culture experiments (Figure 4B and Table 1). 

To assess the underlying cell death mechanism, three chondrosarcoma cell lines (i.e., CH2879, JJ012, and SW1353) were treated with a high dose of romidepsin (i.e., 3 nM) for 24 h or 48 h. Caspase 3/7 activity was significantly induced in SW1353 cells after 24 h, and in all cell lines after 48 h (Figure 4C). A concomitant significant decrease in the viability of JJ012 and SW1353 cells was also observed after 48 h of treatment (Figure 4C). A western blot for cleaved PARP and cleaved caspase 3 confirmed the observed induction of apoptosis (Figure 4D). An increase in apoptosis as well as a reduction in proliferation were also observed in the 3D cell culture models (Figure S3). Cell cycle analysis showed that romidepsin treatment caused a cell cycle arrest in either the G2/M phase for CH2879 or both the G1 phase (24 h) and G2/M phase (48 h) for JJ012 (Figure 4E). Hence, these data indicate that romidepsin could be further explored in vivo as a promising therapeutic strategy for chondrosarcoma, irrespective of the chondrosarcoma subtype and the *IDH* mutation status.

### 2.5. HDAC Inhibitor Combination Drug Screen Identifies Bcl-2 Family Member Inhibitors and Metabolic Compounds as Potential Combination Treatment Strategies

As HDAC inhibitors induce the acetylation of histones, the level of acetylated histone 3 (acH3K9) was determined after 24 h and 48 h treatment with romidepsin. An induction of acH3K9 was observed in all three chondrosarcoma cell lines (Figure 4D). However, this high dose of romidepsin highly affects the cellular growth of these chondrosarcoma cell lines after 72 h (Figure 4A). Lower dosages of romidepsin also showed a prominent induction of acH3K9 (0.75 nM for CH2879 and SW1353 cells and 1 nM for JJ012 cells), whilst the effect on cellular growth was minimal (> 80% nuclei left after 72 h) (Figure S4). These results indicate that low dosages of romidepsin induce changes in epigenetic landscape of chondrosarcoma cells, and these sub-optimal romidepsin dosages could be used to sensitize chondrosarcoma cells to non-epigenetic treatments such as chemotherapy and small molecule inhibitors.

**Figure 5:**
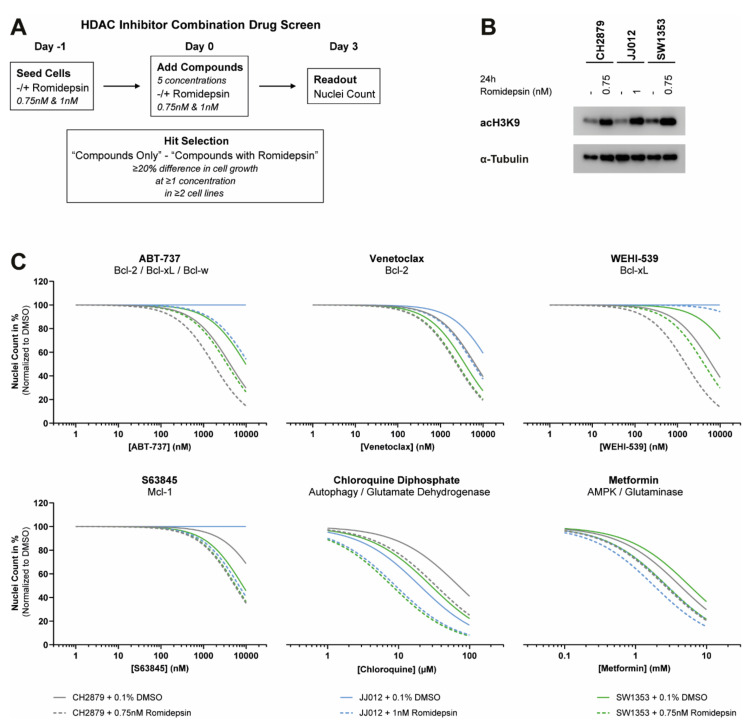
Low dose of romidepsin sensitizes chondrosarcoma cell lines to several non-epigenetic drugs. (**A**) Schematic overview of the performed HDAC inhibitor combination drug screen. (**B**) Western blot for acH3K9 after 24 h treatment with low doses of romidepsin. Histone 3 acetylation was induced in the HDAC inhibitor combination drug screen. α-Tubulin was used as a loading control. Whole blots with densitometry readings can be found in Figure S7B. (**C**) Dose–response curves of the six most promising combination therapies identified in the HDAC inhibitor combination drug screen. Three chondrosarcoma cell lines were treated with twenty non-epigenetic drugs in five concentrations (72 h) with or without romidepsin (96 h). A difference in nuclei count of ≥20% between –/+romidepsin conditions was considered as a potential synergistic treatment combination. Graphs represent the normalized non-linear fit which was calculated based on the individual data points (not shown).

Twenty non-epigenetic drugs were selected for the HDAC inhibitor combination drug screen (Table S4). These drugs either have a known synergistic effect with HDAC inhibitors (e.g., chemotherapy and proteasome inhibitors [22]) or were previously successful as single agent in chondrosarcoma cell lines (e.g., PARP and NAMPT inhibitors [23,24]). A schematic overview of the combination drug screen can be found in Figure 5A. As a screen quality control, the induction of acH3K9 was determined by western blot (Figure 5B). Addition of romidepsin sensitized three chondrosarcoma cell lines to 6 out of 20 non-epigenetic drugs: four Bcl-2 family member inhibitors and two metabolic compounds (Figure 5C). The other 14 compounds did not show a beneficial effect when combined with HDAC inhibition, except for CB-839 treatment in CH2879 cells and sapanisertib treatment in SW1353 cells (Figure S5).

### 2.6. Romidepsin Sensitizes Chondrosarcoma Cells to Bcl-2 Family Member Inhibitors and Affects the Expression of Both Anti- and Pro-Apoptotic Proteins

**Figure 6:**
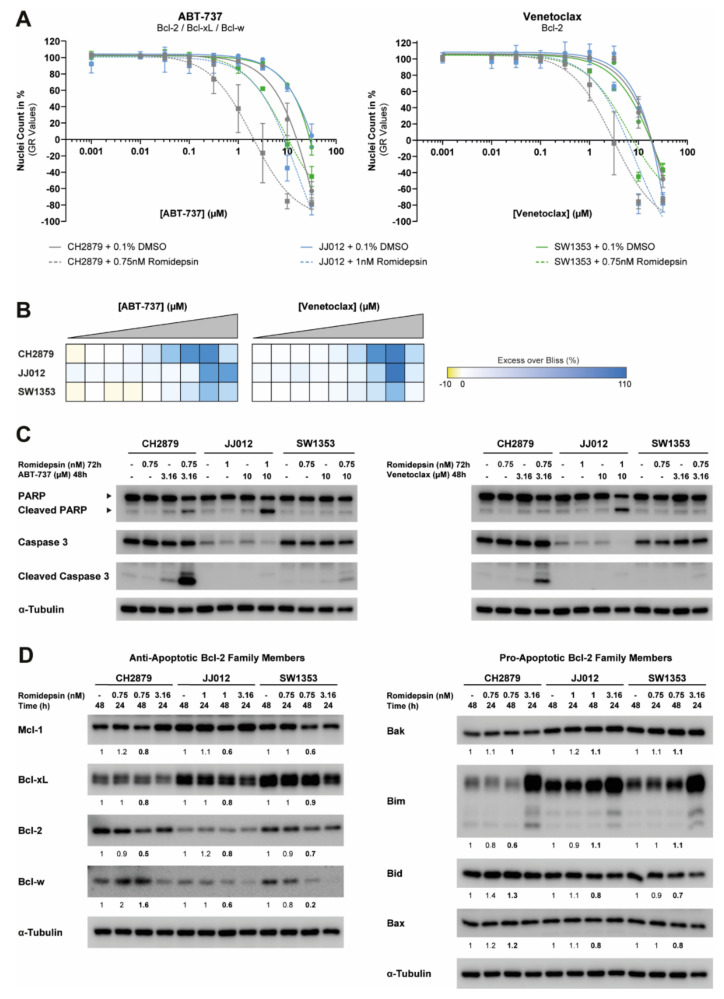
Romidepsin sensitizes chondrosarcoma cells to Bcl-2 family member inhibitors and affects the expression of both anti- and pro-apoptotic proteins. (**A**) Dose–response curves of single or combination treatment strategies after 72 h of treatment for three chondrosarcoma cell lines. Romidepsin sensitized chondrosarcoma cell lines to both ABT-737 and venetoclax treatment. Data were corrected for growth rate (GR values). Data points represent the mean of two experiments performed in triplicate ± standard deviation. (**B**) Heatmaps of the calculated Excess over Bliss scores for the combination treatment strategies. Yellow represents antagonism, white represents additivity and blue represents synergy. Combination treatment strategies were synergistic when high concentrations of ABT-737 and venetoclax were used. (**C**) Western blots for full-length/cleaved PARP and full-length/cleaved caspase 3 after single or combined treatment with romidepsin (72 h) and/or ABT-737/venetoclax (48 h). Combination treatment strategies induced more apoptosis than single agent treatment strategies. α-Tubulin was used as a loading control. Whole blots with densitometry readings can be found in Figure S7C. (**D**) Western blots for anti-apoptotic and pro-apoptotic Bcl-2 family members after 24 h and 48 h treatment with low doses of romidepsin (0.75 nM or 1 nM). Low romidepsin doses affected the expression of both anti- and pro-apoptotic Bcl-2 family members. As a positive control, 24 h treatment with a high dose of romidepsin (3.16 nM) was used. α-Tubulin was used as a loading control. For each sample, expression of Bcl-2 family members was normalized to α-tubulin expression. For each cell line, fold changes were calculated relative to the untreated control. Whole blots with densitometry readings can be found in Figure S7D.

The general Bcl-2 family member inhibitor, ABT-737, and the specific Bcl-2 inhibitor, venetoclax, showed the highest synergistic effect when combined with romidepsin, especially at higher drug concentrations (Figure 6A,B, Figure S6). Induction of apoptosis due to these combination treatments was confirmed by the expression of cleaved PARP and cleaved caspase 3 in both CH2879 and JJ012 cells, but apoptotic cell death was less pronounced in SW1353 cells (Figure 6C). To understand the underlying mechanism, the protein expression of anti- and pro-apoptotic Bcl-2 family members was determined after 24 h to 48 h treatment with a low dose of romidepsin. The expression of all anti-apoptotic family members was downregulated after 48 h of treatment, except for Bcl-w expression in CH2879 cells (Figure 6D). In contrast, the expression of pro-apoptotic proteins was either up- or downregulated after 48 h treatment with low dosages of romidepsin, depending on the pro-apoptotic protein subtype and chondrosarcoma cell line (Figure 6D). Hence, these results indicate that HDAC inhibition can change the balance between pro- and anti-apoptotic proteins, which could explain why the combination treatment with HDAC inhibition and Bcl-2 family member inhibition is highly synergistic in chondrosarcoma cells.

## 3. Discussion

In this study, we explored whether the methylome is altered upon progression from *IDH* mutant enchondroma towards chondrosarcoma. We showed that the CIMP-positive status is retained in high-grade *IDH* mutant chondrosarcoma. Moreover, the number of highly methylated genes seemed to increase upon tumor progression. This suggests that methylation patterns change upon chondrosarcoma progression, and that an increased number of methylated genes is associated with aggressiveness of the disease. Since we chose to include a homogeneous *IDH* mutant subgroup of chondrosarcomas, it remains to be established whether methylation is equally important in *IDH* wildtype chondrosarcomas. The epigenetics compound screen results, indicating sensitivity to the same epigenetic modulators, suggest that the epigenetic landscape is comparable between *IDH* mutant and wildtype chondrosarcomas. This is further supported by the lack of a synthetic lethal interaction between the *IDH* mutation and a broad spectrum of epigenetic regulators, even though these kinds of synthetic lethal interactions (i.e., BET protein and DNMT inhibitors) were previously described in *IDH* mutant acute myeloid leukemia (AML) and glioma [25,26,27]. Together, these data suggest that the epigenetic landscape is highly altered in cartilage tumors, and that these changes go beyond the effect of the *IDH* mutation on the methylome. 

Based on the retained CIMP-positive status and the increasing amount of hypermethylated genes in high-grade *IDH* mutant chondrosarcoma tumors, we expected that targeting of DNMTs by for instance decitabine and azacytidine would be effective in chondrosarcoma. However, none of these DNMT inhibitors had a pronounced effect on chondrosarcoma cell viability. Nevertheless, the epigenetics compound screens identified several other classes of interesting drug targets for both *IDH* wildtype and *IDH* mutant chondrosarcoma, among which inhibitors against Aurora kinases, FLT3, HDAC, and JAK showed the most pronounced effect on chondrosarcoma cell growth. Most of these targets play a major role in growth signaling pathways (FLT3 and JAK) or cell cycle control (Aurora kinases). We have previously shown that targeting cell cycle regulators, especially Checkpoint Kinase 1 (CHK1), could be a promising therapeutic strategy for chondrosarcoma patients [28]. Hence, non-epigenetic targets are of interest, but this went beyond the scope of the present study.

The HDAC enzymes play a prominent role in skeletal development and aberrant expression is associated with a wide variety of bone-related abnormalities. HDAC1 and HDAC3 are most abundantly expressed in healthy human cartilage [29], and we showed that these subtypes are also highly expressed in chondrosarcoma cell lines. Of note, the expression of HDAC2, HDAC7, and HDAC10 seemed to be increased in chondrosarcoma cell lines as compared to healthy human cartilage [29], and romidepsin targets one of these subtypes (i.e., HDAC2). Overexpression of class I HDAC subtypes (HDAC1, -2, -3, and -8) inhibits the expression of cartilage-specific genes (e.g., *COL2A1*), leading to the disruption of normal cartilage development [30], while its inhibition might promote differentiation and reduce proliferation. This mechanism could underlie romidepsin sensitivity in chondrosarcoma, but warrants further investigation. 

Previously, it was shown by other groups that the HDAC inhibitors romidepsin, trichostatin A, and sodium valproate affect cell proliferation in 2D in vitro models of chondrosarcoma [31,32]. In this study, we found that chondrosarcoma cell lines are highly sensitive to HDAC inhibition in both 2D and 3D in vitro models, especially to pan-HDAC inhibitors and the specific class I HDAC inhibitor romidepsin. Moreover, we showed that sensitivity to romidepsin is independent of the *IDH* mutation status and the chondrosarcoma subtype (i.e., central conventional-, dedifferentiated-, or mesenchymal chondrosarcoma), indicating that HDAC inhibition could be a promising therapeutic strategy for all chondrosarcoma patients. Romidepsin is clinically approved for the treatment of cutaneous T-cell lymphoma and other peripheral T-cell lymphomas. In vitro studies of romidepsin treatment in T-cell lymphomas report IC_50_ values in the low nanomolar range (1 to 11 nM) [33,34], which are comparable to the determined GR_50_ and IC_50_ values in our 2D and 3D chondrosarcoma cell culture models. The currently approved dosing regimen of romidepsin in patients with T-cell lymphomas reaches maximum plasma concentrations of 700 nM [35], which further supports that our findings are not related to off-target and toxic side effects. Moreover, romidepsin significantly reduces tumor growth in a subcutaneous chondrosarcoma mouse model [31]. Additional research in an orthotopic chondrosarcoma mouse model [36] should be performed to confirm the efficacy of romidepsin in an *IDH* mutation status independent manner. As in vitro and in vivo models differ in nutrient availability, metabolism and cell types involved in tumor growth, the downstream effects of *IDH* mutations on the epigenetic landscape and the metabolism might also vary between these two types of models.

We also explored if sub-optimal concentrations of romidepsin could be used in a combination treatment strategy, to either sensitize chondrosarcoma to non-epigenetic therapies or to reduce HDAC inhibitor toxicity. However, HDAC inhibition could not sensitize chondrosarcoma cells to four different types of chemotherapy (i.e., aclarubicin, cisplatin, doxorubicin, and temozolomide), although this has been extensively described in other tumor types [22]. Only the combinations of romidepsin with two metabolic compounds (i.e., chloroquine and metformin) and four Bcl-2 family member inhibitors (i.e., ABT-737, venetoclax, WEHI-539, and S63845) were synergistic in chondrosarcoma cell lines. HDAC inhibitors highly alter the metabolic state of glioblastoma cell lines [37], and a similar mechanism in chondrosarcoma could underlie the sensitization to the glutaminolytic pathway inhibitors chloroquine and metformin. However, in breast cancer the synergistic effect between HDAC inhibition and chloroquine has been ascribed to the inhibition of the autophagic flux [38]. The synergistic effect between HDAC inhibition and Bcl-2 family member inhibitors in chondrosarcoma might be caused by an imbalance between pro- and anti-apoptotic Bcl-2 family members. We showed that romidepsin changes the expression levels of these proteins and shifts the balance to a more pro-apoptotic phenotype. Concomitant inhibition of anti-apoptotic Bcl-2 family members will further lower the apoptotic threshold, leading to a more pronounced induction of apoptosis as compared to single agent treatment. Changes in the expression of the pro- and anti-apoptotic Bcl-2 family members due to HDAC inhibition have been observed in several other tumor types [39,40,41]. The exact mechanism underlying the changes in the expression of these proteins is not completely understood, although one study ascribes it to local deacetylation of histone H3 at Bcl-2 promoters [39], suggesting that HDAC inhibitors could regulate the transcriptional activity of Bcl-2 family member genes. The biological mechanism underlying the synergistic effect between HDAC inhibition and glutaminolysis or Bcl-2 family member inhibition in chondrosarcoma warrants further investigation. Since several clinical trials have shown that solid tumors are resistant to HDAC inhibitor monotherapy [42], the identified combination therapies could help to increase the therapeutic potential of both HDAC inhibitors and the small molecule inhibitors in chondrosarcoma.

## 4. Materials and Methods

### 4.1. DNA Methylation Array

Fresh-frozen tumor samples of 20 subjects with solitary benign, low-grade or high-grade cartilage tumors (Table S1) were collected at the LUMC. All samples were handled and coded according to the “Code for Proper Secondary Use of Human Tissue in The Netherlands” (Dutch Federation of Medical Scientific Societies), and their use was approved by the LUMC ethical committee (B17.039). Genomic DNA was isolated with the Wizard Genomic DNA Purification Kit (Promega) according to the manufacturer’s protocol. The *IDH* mutation status (Table S1) was confirmed with Sanger sequencing as previously described [6]. Bisulfite conversion of genomic DNA and the Infinium HumanMethylation450 BeadChip array (Illumina, San Diego, CA, USA) were performed as previously described [19]. CIMP-status of all samples was determined as previously described [6]. Hierarchical clustering was based on the 2000 most variable CpG sites and performed with the Ward’s method. Differentially methylated genes between benign/low-grade and high-grade samples were determined with a global test [43] after Beta MIxture Quantile dilation (BMIQ) normalization [44]. To identify enriched pathways in the differentially methylated gene sets, the ensemble gene set enrichment analyses (EGSEA) was used [45]. 

### 4.2. Compounds

Detailed lists of all 128 compounds included in the epigenetics compound library (L1900, Selleckchem, Houston, TX, USA) and of all twenty compounds included in the custom designed compound library for the HDAC inhibitor combination drug screen are available in the Supplementary Files (Table S3 and Table S4, respectively). The IDH1 mutant (R132H and R132C) inhibitor AGI-5198 (14624, Cayman Chemical, Ann Arbor, MI, USA), the cell permeable derivative of D-2-HG ((2R)-Octyl-α-hydroxyglutarate, 16366, Cayman Chemical), the HDAC inhibitor romidepsin (S3020, Selleckchem), the glutamate dehydrogenase inhibitor chloroquine diphosphate (S4157, Selleckchem), the glutaminase inhibitor metformin HCl (S1950, Selleckchem) and the B-cell lymphoma 2 (Bcl-2) family member inhibitors ABT-737 (Bcl-2/Bcl-xL/Bcl-w, S1002, Selleckchem), S63845 (Mcl-1, S8383, Selleckchem), venetoclax (Bcl-2, S8048, Selleckchem), and WEHI-539 (Bcl-xL, A3935, APExBIO, Houston, TX, USA) were dissolved in DMSO, PBS, or RPMI 1640 medium according to the manufacturer’s protocol.

### 4.3. Cell Culture

The central conventional chondrosarcoma cell lines CH2879 (*IDH* wild type (*IDH*^WT^)) [6,46], JJ012 (*IDH1*^R132G^) [6,47], SW1353 (ATCC, *IDH2*^R172S^) [6], CH3573 (*IDH*^WT^) [48] and L835 (*IDH1*^R132C^) [6,49]; the dedifferentiated chondrosarcoma cell lines NDCS1 (*IDH*^WT^) [6,50], HT1080 (*IDH1*^R132C^) [19,51], L2975 (*IDH2*^R172W^) [6,49] and L3252B (*IDH*^WT^) [49]; and the mesenchymal chondrosarcoma cell line MSC170 (*IDH*^WT^) [52] were cultured as described previously [23]. Long-term AGI-5198 treated cell lines (20 passages with 1.5 µM AGI-5198) [19] were cultured in RPMI 1640 medium (Gibco, Invitrogen Life-Technologies, Scotland, UK) supplemented with 10% heat-inactivated fetal bovine serum (FBS) (F7524, Sigma-Aldrich, Saint Louis, MO, USA) and 10µM AGI-5198 (to minimize D-2-HG production). The 3D cell cultures were established and cultured as previously described [53]. All cells were cultured in a humidified incubator with 5% CO_2_ at 37 °C. PCR-based mycoplasma tests and STR profiling (GenePrint 10 System, Promega, Madison, WI, USA) were performed regularly.

### 4.4. Primary Epigenetics Compound Screen

The chondrosarcoma cell lines CH2879, JJ012, and SW1353 were seeded in optimized cell densities (5000, 3000, and 3000/well, respectively) in black 96-well plates. After overnight attachment, cells were treated with the epigenetics compound library at a concentration of 2 µM. After 72 h of treatment, cells were fixed with 4% formaldehyde and stained with 2 µg/mL Hoechst 33342 (H1399, Invitrogen Life-Technologies). Plates were imaged using a BD Pathway 855 microscope (BD Biosciences, Breda, The Netherlands) and the number of Hoechst 33342 positive nuclei was quantified using the Image-Pro software (Media Cybernetics, Rockville, MD, USA). All plates were included in the analysis and data were normalized to the negative controls (i.e., PBS and DMSO) to obtain percent of control values. All compounds that reduced the nuclei count ≥50% in at least one of the cell lines were selected for the secondary epigenetics compound screen. The screen was performed in triplicate. A schematic overview of the primary epigenetics compound screen can be found in Figure 2A. A list of all compounds, including the numbering and specific targets, can be found in Table S3.

### 4.5. Secondary Epigenetics Compound Screen

To reduce the D-2-HG levels, *IDH1* mutant cell lines (i.e., JJ012, HT1080, and L835) were pre-exposed to 10 µM AGI-5198 for 72 h [19]. Subsequently, the *IDH1* mutant cell lines were seeded in black 96-well plates (3000, 5000, and 10000/well, respectively) in normal growth medium supplemented with 10µM AGI-5198 or 0.1% DMSO as a control. To mimic D-2-HG production, CH2879 cells were seeded (5000/well) in normal growth medium supplemented with 250 µM D-2-HG or 1% PBS as a control. After overnight attachment, cells were treated with the selected compounds (*n* = 61) from the primary screen at a concentration of 2 µM. Compounds were diluted in medium supplemented with 0.1% DMSO, 10µM AGI-5198, 1% PBS or 250 µM D-2-HG. After 72 h of treatment, a nuclei count was performed as described under the methods section of the primary epigenetics compound screen. All plates were included in the analysis and data were normalized to the negative controls (i.e., PBS and DMSO) to obtain percent of control values. Compounds that reduced the nuclei count ≥50% in all cell lines were considered as potential therapeutic options for both *IDH* wildtype and *IDH* mutant chondrosarcomas. A ≥75% difference in nuclei count between cells treated with or without 10 µM AGI-5198 or cells treated with or without 250 µM D-2-HG was considered as a potential synthetic lethal interaction with the *IDH* mutation. The screen was performed in triplicate. A schematic overview of the secondary epigenetics compound screen can be found in Figure 2C. 

### 4.6. HDAC Inhibitor Combination Drug Screen

CH2879 cells (7000/well), JJ012 cells (3000/well), and SW1353 cells (3000/well) were seeded in 96-well plates in normal growth medium supplemented with 0.1% DMSO or romidepsin (1 nM (JJ012 cells) and 0.75 nM (CH2879 and SW1353 cells)). After overnight attachment, cells were treated with the custom designed compound library. For each library compound, five concentrations were tested, which were based on previous experience (Table S4). Compounds were diluted in normal growth medium supplemented with 0.1% DMSO or romidepsin (0.75 nM or 1 nM) and added to the cells. The solvents DMSO and PBS were used as negative controls and 3.16 nM romidepsin was used as positive control. After 72 h of treatment, cells were fixed with 4% formaldehyde and stained with 2 µg/mL Hoechst 33342. To automatically count the nuclei, the Cellomics ArrayScan VTI HCS 700 series and HCS Studio Cell Analysis Software (ThermoFisher Scientific, Waltham, MA, USA) were used. Plates were excluded from the analysis if a low correlation with the other replicates was observed (R square ≤ 0.8) combined with a Z’-factor < 0.5. Data were normalized to the negative controls (i.e., PBS and DMSO) to obtain percent of control values. A ≥20% difference in nuclei count between cells treated with or without romidepsin was considered as a potential synergistic treatment combination. The screen was performed in triplicate. A schematic overview of the HDAC inhibitor combination drug screen can be found in Figure 5A. A list of all compounds, including the specific targets, can be found in Table S4.

### 4.7. Cell Viability and Nuclei Count Assays

For the 2D cell cultures, the cell viability assays, nuclei count assays and data analysis were performed as previously described [23]. Cells were treated for 72 h with tubacin, romidepsin, ABT-737, venetoclax, WEHI-539, S63845, chloroquine diphosphate, or metformin in concentrations ranging from 0.0316 nM to 31.6 mM. Cells treated with DMSO or PBS were used as a negative control. Experiments were performed in triplicate and repeated one to three times.

For the 3D cell cultures, the cell viability assays and the immunohistochemical stains (Ki-67 and cleaved caspase 3) were performed as previously described [53]. Cells were grown in alginate beads for 14 days and subsequently treated for 72 h with romidepsin in concentrations ranging from 0.1 to 10 nM. Experiments were performed in triplicate and repeated four times.

### 4.8. siRNA Transfection

Reverse siRNA transfection was performed for CH2879 cells (10,000/well) and JJ012 cells (5000/well) in 96-well plates. To achieve transient knockdown of all HDAC subtypes, cells were transfected with 0.1 µL/well DharmaFECT 3 (Dharmacon, Lafayette, CO, USA) and 50 nM siRNA SMARTpools (Dharmacon), which consist of four individual siRNAs targeting a gene of interest. The mock condition, the siRNA SMARTpool against Glyceraldehyde-3-Phosphate Dehydrogenase *(GAPDH)* (Dharmacon) and an siKinasePool (a mixture of diluted siRNAs targeting kinases with a final total siRNA concentration of 50 nM) were used as negative controls. siRNA SMARTpools against Kinesin Family Member 11 *(KIF11)* and Polo Like Kinase 1 *(PLK1)* (Dharmacon) were used as positive controls. To reduce toxicity, the medium was refreshed 24 h after the transfection. Three days after medium refreshment, cells were fixed, stained with Hoechst 33342 and imaged with the Cellomics ArrayScan VTI HCS 700. Data were normalized to the negative controls to obtain percent of control values. The experiment was performed in triplicate. 

### 4.9. Next Generation RNA Sequencing Analysis

RNA expression of all HDAC subtypes in chondrosarcoma cell lines was extracted from a next generation RNA sequencing dataset we previously described [19]. Reads per kilobase per million (RPKM) were used to describe gene expression levels.

### 4.10. Apoptosis and Cell Cycle Assays

The apoptosis and cell cycle assays were performed and analyzed as previously described [23]. Cells were treated with 3 nM romidepsin for 24 h and 48 h. In both assays, DMSO was used as a negative control. In the apoptosis assay, 5µM ABT-737 combined with 1 µM doxorubicin was used as a positive control. Experiments were performed in singular (cell cycle) or duplicate (apoptosis) and repeated three times.

### 4.11. Western Blotting

Sample preparation, western blotting and quantification were performed as previously described [23]. Cells were treated with different compounds (i.e., romidepsin, ABT-737, and venetoclax) for 24 h to 72 h in concentrations ranging from 0.316 nM to 10 µM. Western blots were stained for the expression of full-length/cleaved caspase 3 (1:1000, 8G10, Cell Signaling Technology (CST), Leiden, The Netherlands), full-length/cleaved PARP (1:1000, 46D11, CST), acetyl-Histone H3 (Lys9) (acH3K9) (1:1000, clone C5B11, CST), Mcl-1 (1:1000, #4572, CST), Bcl-xL (1:1000, 54H6, CST), Bcl-2 (1:1000, D55G8, CST), Bcl-w (1:1000, 31H4, CST), Bak (1:1000, D4E4, CST), Bim (1:1000, C34C5, CST), Bid (1:1000, #2002, CST), and Bax (1:1000, D2E11, CST). α-Tubulin (1:30,000, DM1A, Sigma-Aldrich) was used as a loading control.

### 4.12. Statistical Analysis

To determine significant changes between experimental groups, a nonparametric Kruskal–Wallis test followed by a Dunn’s post-hoc test was performed. A Grubbs’ test was performed to detect outlier values in the dose–response curve datasets. All statistical tests were performed in GraphPad Prism 8. The Z’-factor was used as a measure for compound screen quality and was calculated to determine the effect size between negative and positive controls [54]. To calculate synergy in combined drug treatments, the Bliss independence model was used [55,56]. Heatmap figures were created with the online tool MORPHEUS (Broad Institute, Cambridge, MA, USA).

## 5. Conclusions

In summary, alterations in the methylome of *IDH* mutant cartilage tumors are associated with tumor progression and this study shows that targeting of epigenetic regulators could be a potential therapeutic strategy for both *IDH* wildtype and *IDH* mutant chondrosarcoma patients. Our study establishes that HDAC enzymes play a prominent role in the epigenetic landscape and survival of chondrosarcoma cells, especially class I HDACs. Chondrosarcoma cell lines are highly sensitive to the class I HDAC inhibitor romidepsin, irrespective of the chondrosarcoma subtype or the *IDH* mutation status. In addition, our study identifies glutaminolysis and Bcl-2 family member inhibitors as potential candidates to be used in combination with HDAC inhibition, suggesting that romidepsin influences the metabolic state and apoptotic threshold of chondrosarcoma cells. Further studies are needed to elucidate the exact mechanisms underlying HDAC inhibitor sensitivity in chondrosarcoma and to confirm romidepsin efficacy in an orthotopic chondrosarcoma mouse model. Taken together, pharmacological inhibition of HDAC enzymes may represent a promising targeted therapeutic strategy for chondrosarcoma patients. In spite of the fact that the mechanisms underlying HDAC inhibitor sensitivity are currently unknown, the identification of novel treatment strategies for chondrosarcoma remains an important objective, especially for patients with unresectable or high-grade tumors.

## Figures and Tables

**Table 1:** IC_50_ and growth corrected values (i.e., GR_50_) of 72 h romidepsin treatment in chondrosarcoma cell lines

Cell Line	Subtype	*IDH* Status	2D Cell Culture	3D Cell Culture	
			GR_50_ (nM)	IC_50_ (nM)	IC_50_ (nM)
MCS170	Mesenchymal	Wildtype	0.89	2.69	-
L835	Central conventional	*IDH1* R132C	0.97	29.2	-
JJ012 + AGI-5198	Central conventional	*IDH1* R132G (inhibited)	1.06	0.96	-
SW1353	Central conventional	*IDH2* R172S	1.10	1.01	2.00
HT1080	Dedifferentiated	*IDH1* R132C	1.10	0.99	-
CH3573	Central conventional	Wildtype	1.26	1.37	-
L2975	Dedifferentiated	*IDH2* R172W	1.27	1.29	-
L3252B	Dedifferentiated	Wildtype	1.30	1.51	-
CH2879	Central conventional	Wildtype	1.79	1.61	2.96
NDCS1	Dedifferentiated	Wildtype	1.92	1.57	-
JJ012	Central conventional	*IDH1* R132G	1.96	1.71	5.08

## Supplementary Materials

The following are available online at https://www.mdpi.com/2072-6694/12/12/3589/s1, Figure S1: Hypermethylation keeps increasing even within high-grade *IDH* mutant chondrosarcomas and mainly affects signal transduction and inflammation related genes, Figure S2: Drug screening does not identify a synthetic lethal interaction between epigenetic regulators and *IDH* mutations, Figure S3: Romidepsin inhibits cell proliferation and induces apoptosis in 3D cell cultures of chondrosarcoma cell lines, Figure S4: Low dose of romidepsin induces the level of histone 3 acetylation in chondrosarcoma cell lines, whilst cell viability is minimally affected, Figure S5: A selection of non-epigenetic drugs does not exhibit synergy in combination with a low dose of romidepsin, Figure S6: Romidepsin sensitizes chondrosarcoma cells to Bcl-2 family member inhibitors and metabolic compounds, Figure S7: Whole blots with densitometry readings from the performed western blots, Table S1: Characteristics of patient samples that were used for the DNA methylation array, Table S2: Significantly differentially methylated genes in benign/low-grade and high-grade cartilage tumors, Table S3: Detailed list of all compounds included in the epigenetics compound library (L1900, Selleckchem), Table S4: Detailed list of all the compounds included in the custom designed compound library for the HDAC inhibitor combination drug screen.
