# Supplementary material for: Beyond the Influence of IDH Mutations: Exploring Epigenetic Vulnerabilities in Chondrosarcoma
Source: Cancers (Basel). 2020 Nov 30;12(12):3589. doi: 10.3390/cancers12123589 (PMC7760027; doi:10.3390/cancers12123589)
Supplement: Supplementary file 1 [file cancers-12-03589-s001.pdf]

Supplementary Materials

Beyond the Influence of *IDH* Mutations: Exploring Epigenetic Vulnerabilities in Chondrosarcoma

Sanne Venneker, Alwine B. Kruisselbrink, Zuzanna Baranski, Ieva Palubeckaite, Inge H. Briaire-de Bruijn, Jan Oosting, Pim J. French, Erik H.J. Danen and Judith V.M.G. Bovée

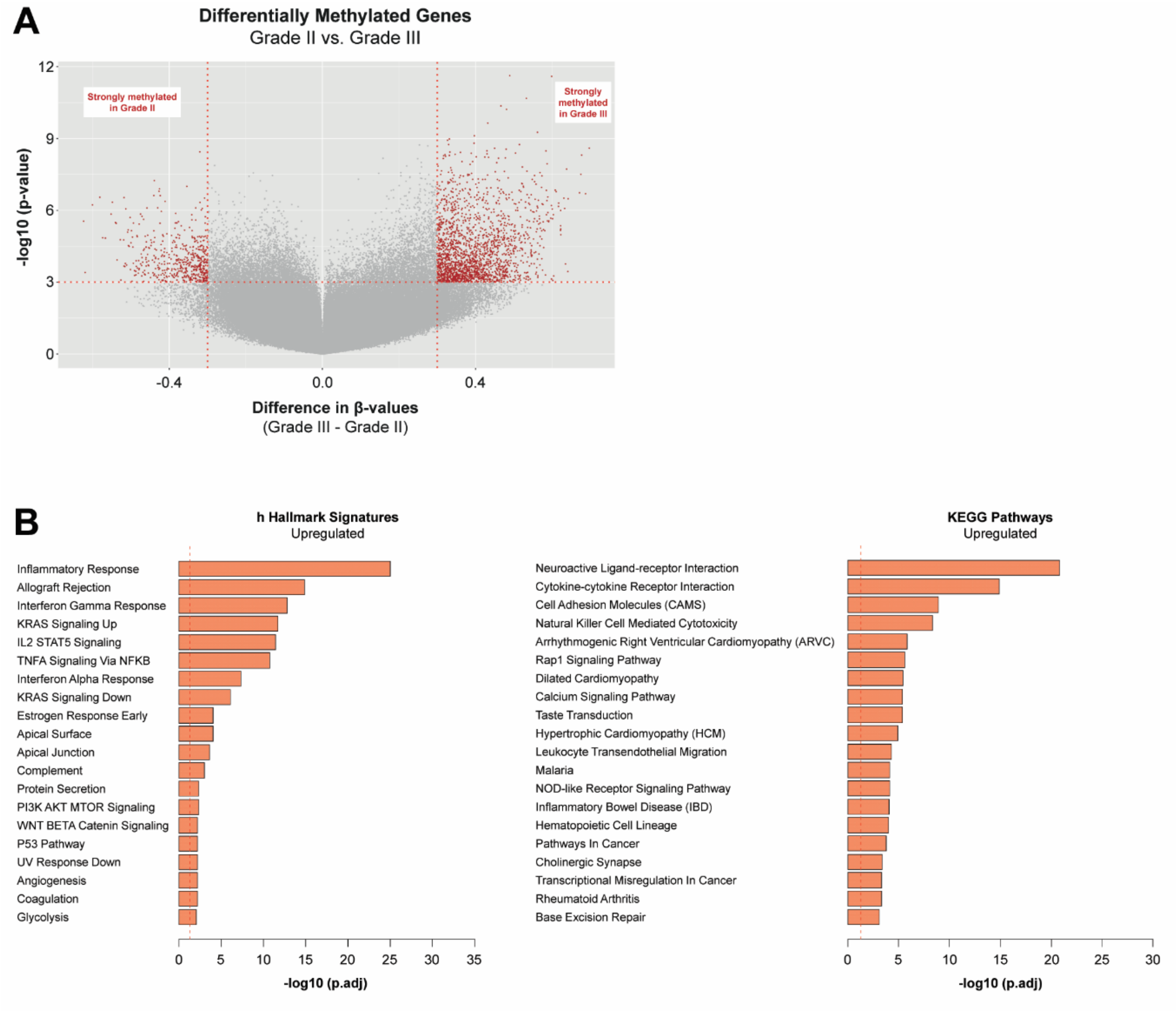

Figure S1. Hypermethylation keeps increasing even within high-grade *IDH* mutant chondrosarcomas and mainly affects signal transduction and inflammation related genes. (**A**) Volcano plot of differentially methylated genes between chondrosarcoma grade II and grade III tumors. Vertical red lines indicate a difference in β-values of at least 0.3 between the two groups. Significantly differentially methylated genes are indicated with red (cut-off at $p < 0.001$). (**B**) EGSEA analysis performed on the significantly differentially methylated gene sets. Both h Hallmark signatures and KEGG pathways identified that mainly genes related to signal transduction and inflammation were affected.

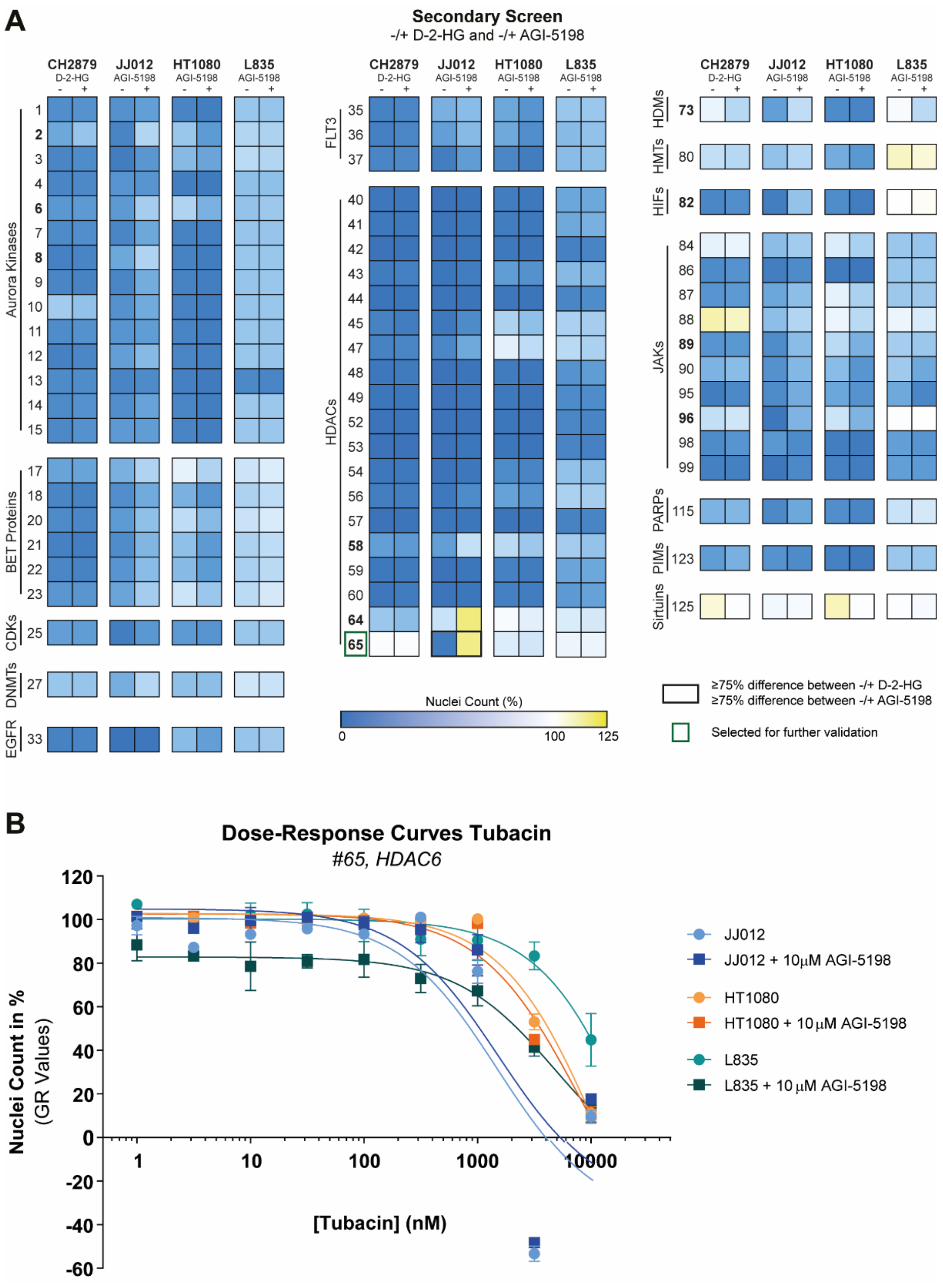

Figure S2. Drug screening does not identify a synthetic lethal interaction between epigenetic regulators and *IDH* mutations. (**A**) Heatmaps of the results from the secondary epigenetics compound screen, in which blue indicates growth inhibition and yellow growth induction. Five chondrosarcoma cell lines –/+ 10 µM AGI-5198 or –/+ 250 µM D-2-HG were treated with 61 compounds at a concentration of 2 µM for 72 h. One potential synthetic lethal interaction (≥75% difference between with or without AGI-5198 treatment) was observed in the JJ012 cell line. (**B**) Dose-response curves of compound #65 (tubacin), a specific HDAC6 inhibitor for three *IDH1* mutant chondrosarcoma cell lines. Short-term treatment with 10 µM AGI-5198 (i.e., 72 h pre-exposure) did not rescue the effect of tubacin in all three cell lines. Dose-response curves were corrected for growth rate (GR values). Data points represent the mean of one experiment performed in triplicate ± standard deviation.

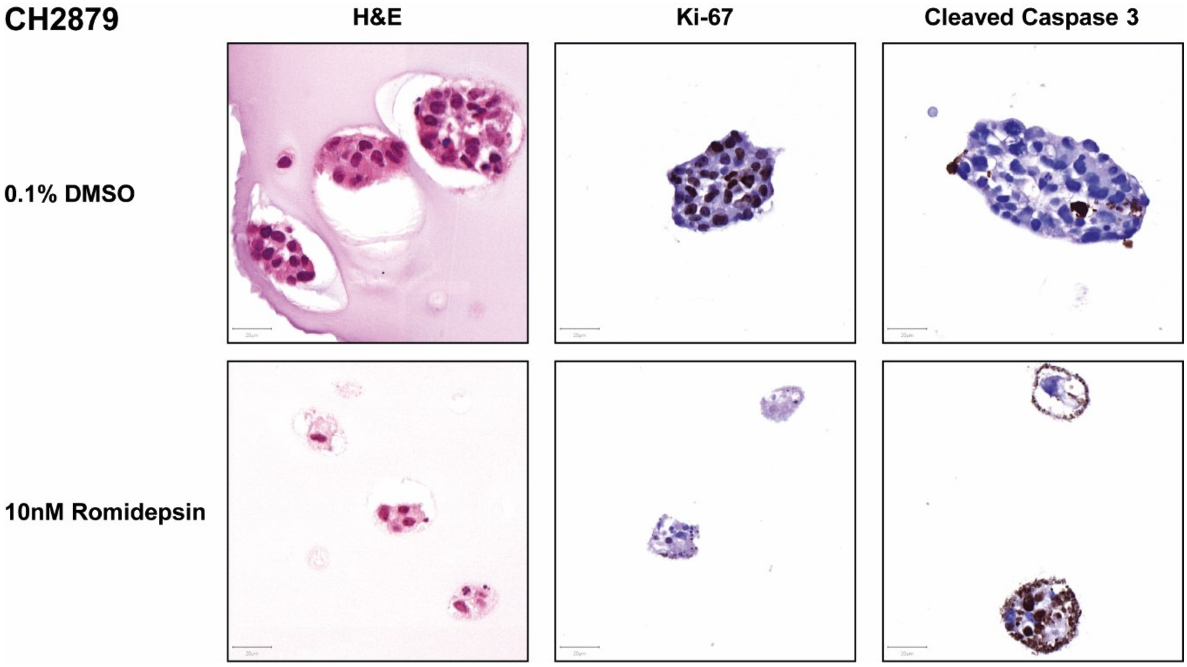

Figure S3. Romidepsin inhibits cell proliferation and induces apoptosis in 3D cell cultures of chondrosarcoma cell lines. Haematoxylin and Eosin (H&E), Ki-67, and cleaved caspase 3 stains performed on CH2879 spheroids after 72 h of treatment with 0.1% DMSO or 10 nM romidepsin. As compared to the control, romidepsin inhibits proliferation (reduced Ki-67 levels and smaller spheroids) and induces apoptosis (increased cleaved caspase 3 levels) in CH2879 spheroids. Similar observations were made for JJ012 and SW1353 spheroids. Scale bar: 20 µm.

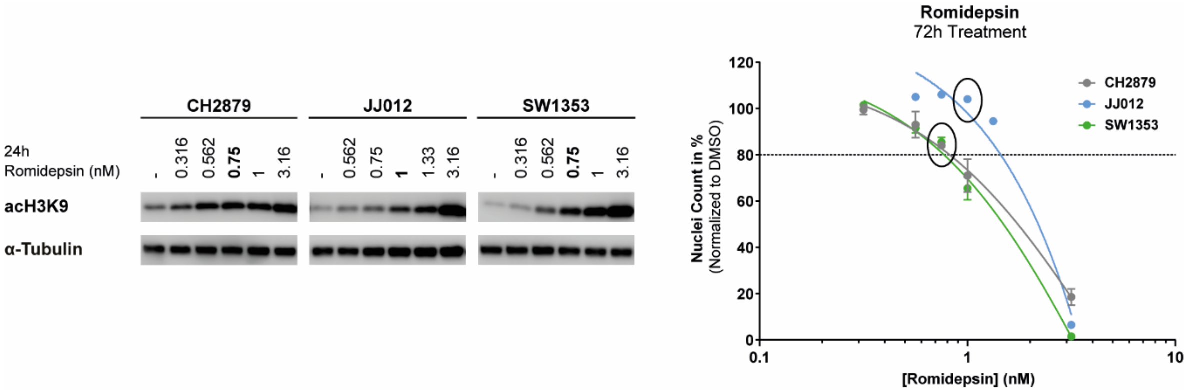

Figure S4. Low dose of romidepsin induces the level of histone 3 acetylation in chondrosarcoma cell lines, whilst cell viability is minimally affected. Western blot for acH3K9 after 24 h treatment with low dosages of romidepsin (0.316 to 1.33 nM) and the corresponding cell viability after 72 h of treatment with these specific concentrations. Low romidepsin doses induced acH3K9 and showed a minimal effect on cell growth (>80% nuclei left). As a positive control, 24 h treatment with a high dose of romidepsin (3.16 nM) was used. α-Tubulin was used as a loading control. Whole blots with densitometry readings can be found in Figure S7E.

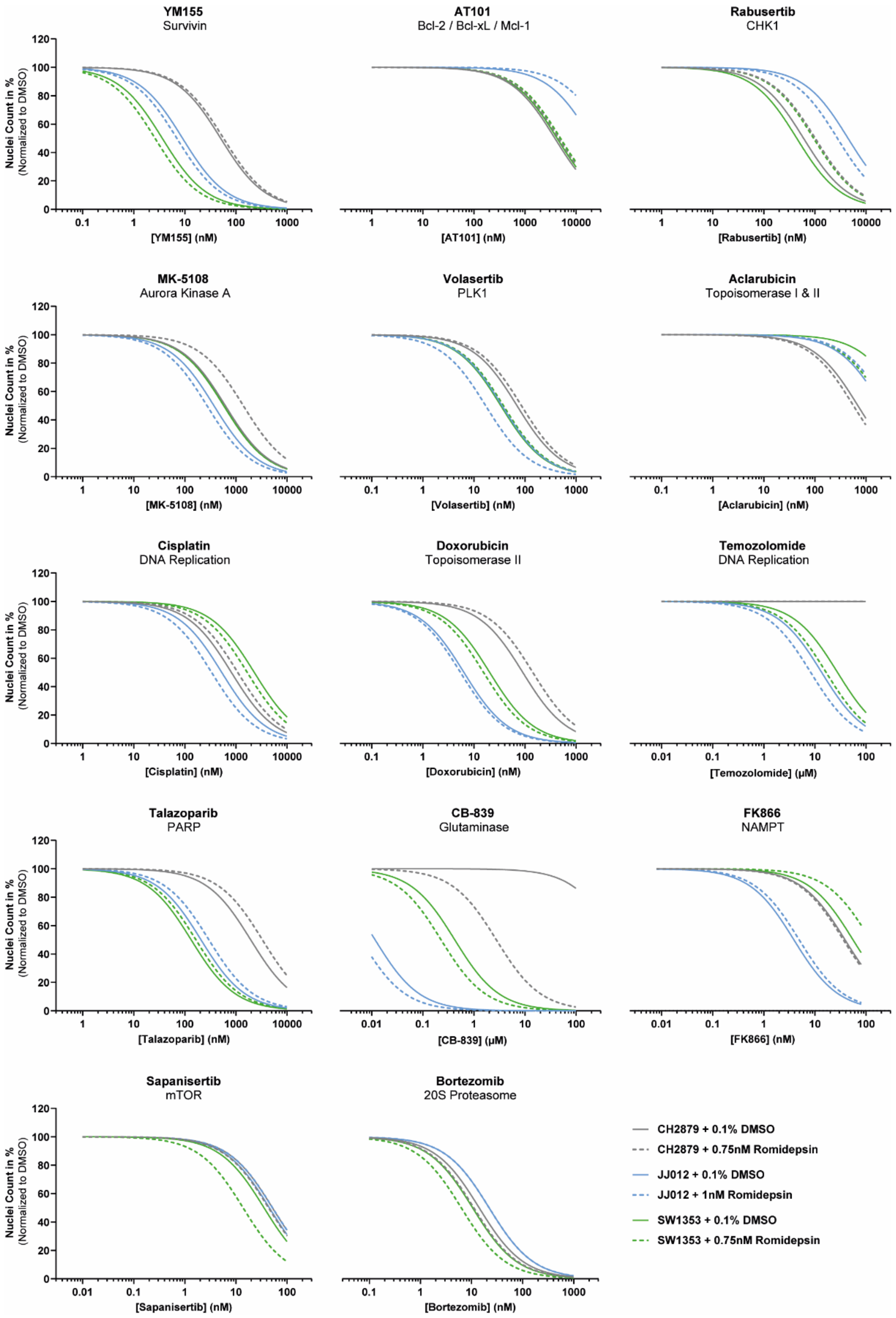

Figure S5. A selection of non-epigenetic drugs does not exhibit synergy in combination with a low dose of romidepsin. Dose-response curves of the fourteen combination therapies that were not considered as a hit in in the HDAC inhibitor combination drug screen. Three chondrosarcoma cell lines were treated with twenty different non-epigenetic drugs in five concentrations (72 h) with or without romidepsin (96 h). A difference in nuclei count of ≥20% between –/+ romidepsin conditions was considered as a potential synergistic treatment combination. Graphs represent the normalized non-linear fit which was calculated based on the individual data points.

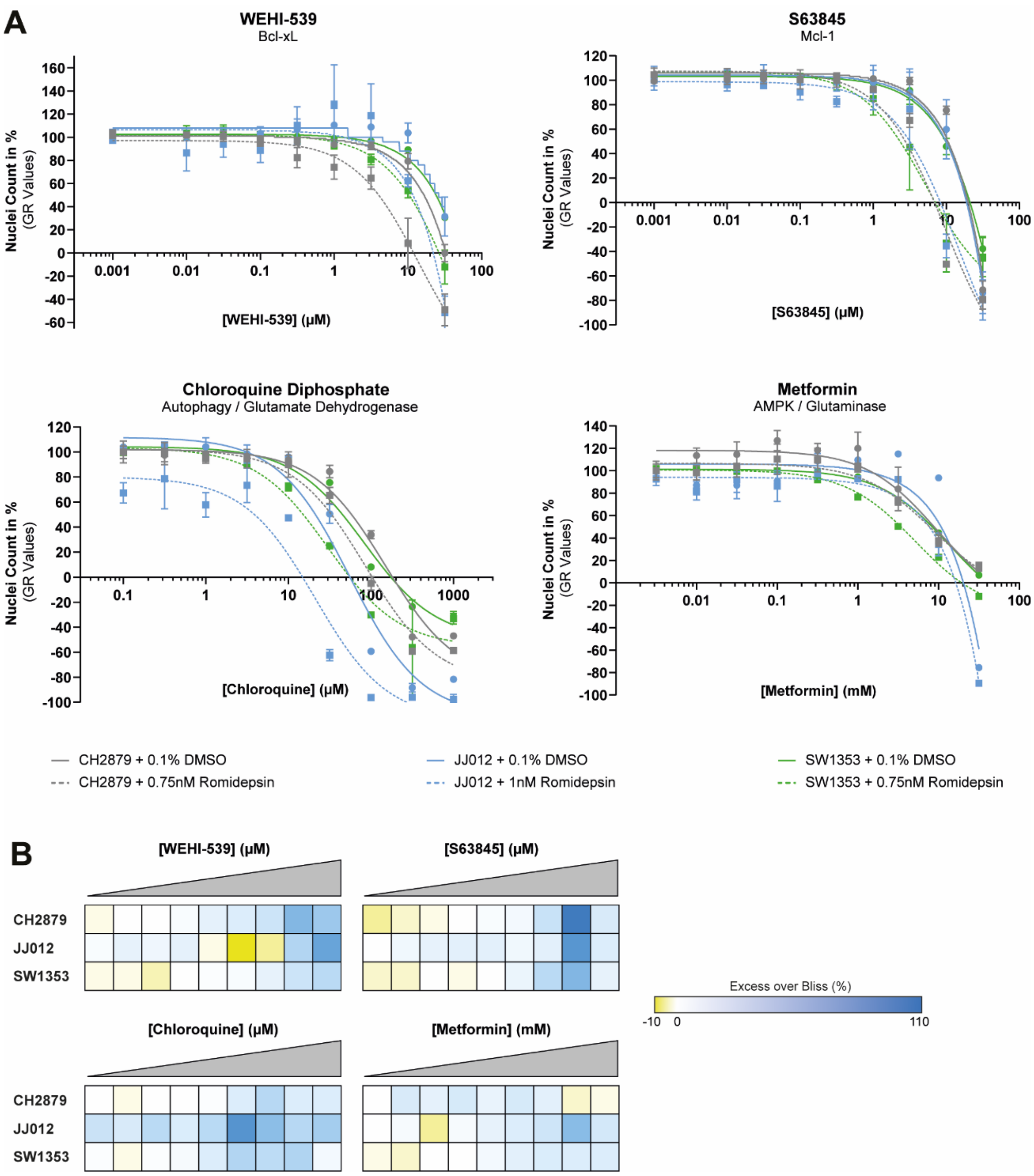

Figure S6. Romidepsin sensitizes chondrosarcoma cells to Bcl-2 family member inhibitors and metabolic compounds. (**A**) Dose-response curves of single or combination treatment strategies after 72 h of treatment for three chondrosarcoma cell lines. Romidepsin treatment sensitized three chondrosarcoma cell lines to WEHI-539, S63845, chloroquine diphosphate, and metformin HCl. Data were corrected for growth rate (GR values). Data points represent the mean of one (i.e., chloroquine and metformin) or two (i.e., WEHI-539 and S63845) experiments performed in triplicate ± standard deviation. (**B**) Heatmaps of the calculated Excess over Bliss scores for the combination treatment strategies. Yellow represents antagonism, white represents additivity and blue represents synergy. All combination treatment strategies were synergistic, but synergy was less pronounced as compared to the combination treatments with ABT-737 and venetoclax.

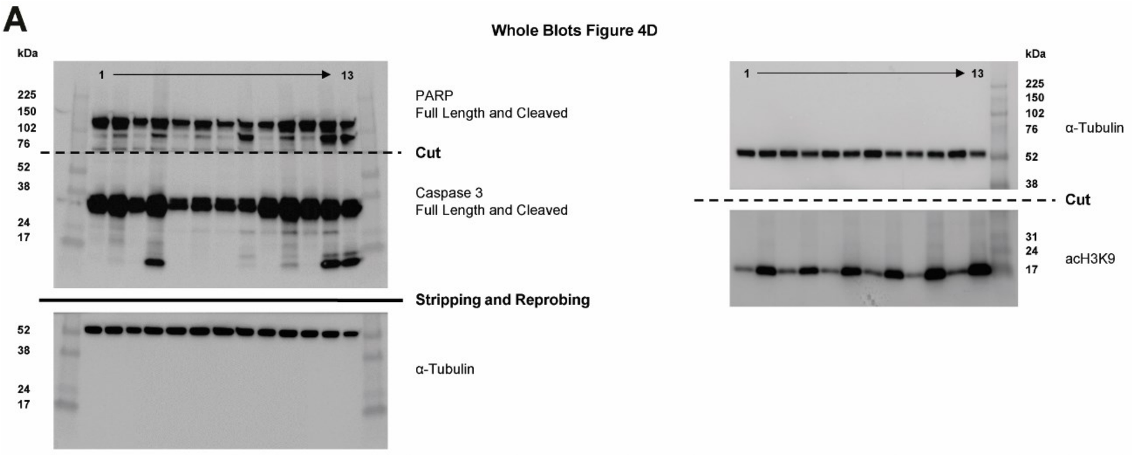

				α-Tubulin	PARP (full)		PARP (cleaved)		Caspase 3 (full)		Caspase 3 (cleaved)	
	Romidepsin			Intensity	Intensity	Ratio	Intensity	Ratio	Intensity	Ratio	Intensity	Ratio
1	CH2879	-	24h	9800832	4919680	0.502	178016	0.018	3875424	0.395	117024	0.012
2		+	24h	10394242	5751066	0.553	781150	0.075	5604764	0.539	1275309	0.123
3		-	48h	6695388	2668316	0.399	105672	0.016	2277884	0.340	196868	0.029
4		+	48h	10303788	4011678	0.389	990132	0.096	5681795	0.551	9402715	0.913
5	JJ012	-	24h	11696816	2774046	0.237	353727	0.030	1277570	0.109	175490	0.015
6		+	24h	13182397	3308415	0.251	698115	0.053	1905948	0.145	532116	0.040
7		-	48h	12960010	2182560	0.168	301344	0.023	1636288	0.126	75036	0.006
8		+	48h	10555479	1311024	0.124	1079067	0.102	1591590	0.151	1063788	0.101
9	SW1353	-	24h	9863139	2687520	0.272	294789	0.030	5200265	0.527	473445	0.048
10		+	24h	10778922	4904394	0.455	953502	0.088	7451961	0.691	3376329	0.313
11		-	48h	9743296	4976192	0.511	559680	0.057	6107360	0.627	336032	0.034
12		+	48h	8195488	3135396	0.383	1691349	0.206	5095794	0.622	13040055	1.591
13	CH2879	Pos. Ctrl	24h	4819787	1735597	0.360	1607071	0.333	3018784	0.626	12392626	2.571

				α-Tubulin	acH3K9	
	Romidepsin			Intensity	Intensity	Ratio
1	CH2879	-	24h	5110155	3496995	0.684
2		+	24h	5646520	9744388	1.726
3		-	48h	5470569	3962088	0.724
4		+	48h	4383351	7398409	1.688
5	JJ012	-	24h	5387109	3571932	0.663
6		+	24h	4401631	9895375	2.248
7		-	48h	7037440	4712480	0.670
8		+	48h	4011680	9377880	2.338
9	SW1353	-	24h	3678210	3243753	0.882
10		+	24h	4520518	13424384	2.970
11		-	48h	6265948	5734260	0.915
12		+	48h	3411130	15556556	4.561

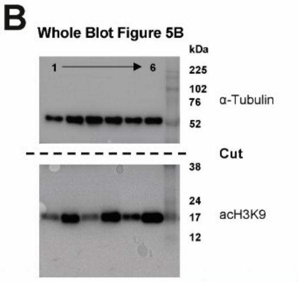

				α-Tubulin	acH3K9	
	Romidepsin			Intensity	Intensity	Ratio
1	CH2879	-	24h	2652756	4013620	1.513
2		+	24h	5033898	9442833	1.876
3	JJ012	-	24h	5385440	4264560	0.792
4		+	24h	4921520	11272548	2.290
5	SW1353	-	24h	3760643	5939506	1.579
6		+	24h	4610676	17034069	3.694

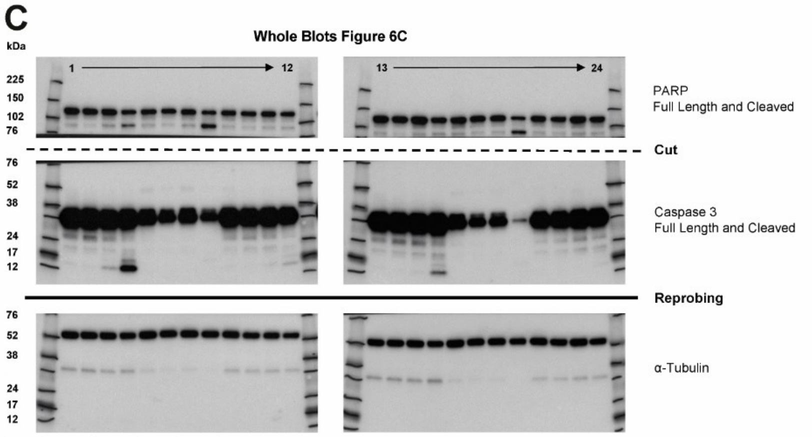

		Romidepsin	ABT-737	α-Tubulin Intensity	PARP (full) Intensity	PARP (full) Ratio	PARP (cleaved) Intensity	PARP (cleaved) Ratio	Caspase 3 (full) Intensity	Caspase 3 (full) Ratio	Caspase 3 (cleaved) Intensity	Caspase 3 (cleaved) Ratio
1	CH2879	-	-	12606282	3625536	0.288	280160	0.022	2326632	0.185	111020	0.009
2		+	-	11559156	3125327	0.270	160673	0.014	2480800	0.215	60544	0.005
3		-	+	10947371	3160760	0.289	396459	0.036	1930897	0.176	526647	0.048
4		+	+	8636055	1926080	0.223	730592	0.085	2071904	0.240	4711210	0.546
5	JJ012	-	-	14050048	2414464	0.172	327072	0.023	738784	0.053	29888	0.002
6		+	-	11244608	2210858	0.197	193595	0.017	389391	0.035	13134	0.001
7		-	+	12470150	2574767	0.206	353958	0.028	580958	0.047	6698	0.001
8		+	+	10515846	1227352	0.117	1096687	0.104	239943	0.023	69564	0.007
9	SW1353	-	-	10857184	2329802	0.215	236785	0.022	1766752	0.163	42592	0.004
10		+	-	8432896	2122290	0.252	192090	0.023	1621664	0.192	138624	0.016
11		-	+	8790012	2469181	0.281	262291	0.030	1721275	0.196	62558	0.007
12		+	+	6923328	1796811	0.260	256157	0.037	1468128	0.212	344645	0.050

		Romidepsin	Venetoclax	α-Tubulin Intensity	PARP (full) Intensity	PARP (full) Ratio	PARP (cleaved) Intensity	PARP (cleaved) Ratio	Caspase 3 (full) Intensity	Caspase 3 (full) Ratio	Caspase 3 (cleaved) Intensity	Caspase 3 (cleaved) Ratio
13	CH2879	-	-	11221224	4034464	0.360	330592	0.029	2303961	0.205	105000	0.009
14		+	-	11459232	3619715	0.316	176390	0.015	2441536	0.213	94944	0.008
15		-	+	12271360	4741760	0.386	328135	0.027	2373856	0.193	90750	0.007
16		+	+	8971865	2871520	0.320	348160	0.039	2947626	0.329	1106088	0.123
17	JJ012	-	-	14295334	3591072	0.251	374272	0.026	657162	0.046	33216	0.002
18		+	-	11651776	3290619	0.282	258881	0.022	362880	0.031	9504	0.001
19		-	+	11643485	3030622	0.260	280054	0.024	380544	0.033	7956	0.001
20		+	+	8562213	1136243	0.133	853058	0.100	65892	0.008	60687	0.007
21	SW1353	-	-	12110752	2940774	0.243	324655	0.027	1355112	0.112	62976	0.005
22		+	-	10381408	2382510	0.229	305730	0.029	1391456	0.134	61504	0.006
23		-	+	12666423	3903613	0.308	394258	0.031	1880398	0.148	61256	0.005
24		+	+	9162720	2714342	0.296	332920	0.036	1704120	0.186	59395	0.006

D

Whole Blots Figure 6D

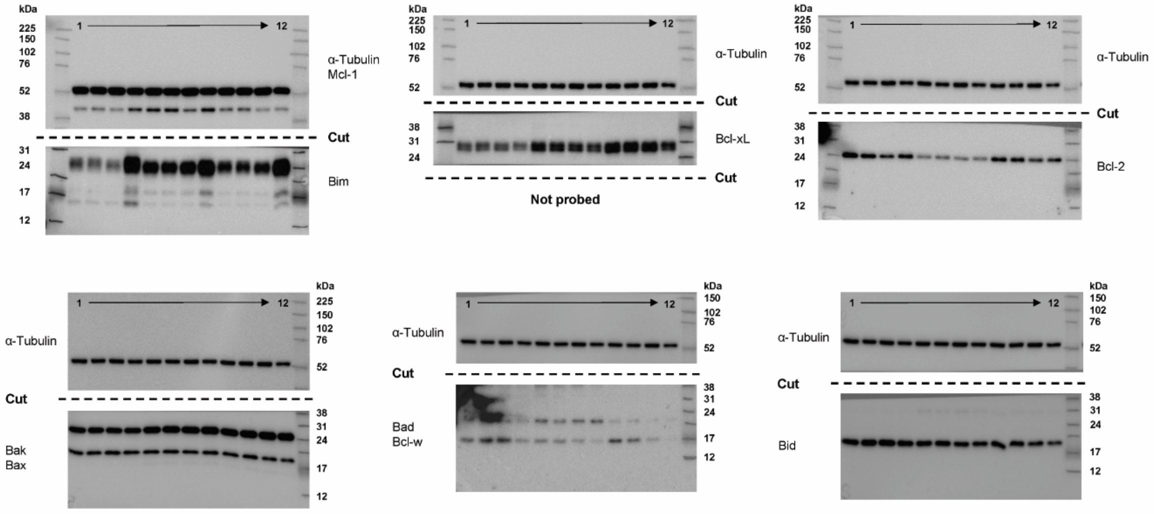

	Romidepsin			α-Tubulin Intensity	Mcl-1 Intensity	Mcl-1 Ratio	Bim Intensity	Bim Ratio	α-Tubulin Intensity	Bcl-xL Intensity	Bcl-xL Ratio	α-Tubulin Intensity	Bcl-2 Intensity	Bcl-2 Ratio
1	CH2879	-	48h	11238045	4113400	0.366	3226509	0.287	13391274	8489760	0.634	11733813	1474240	0.126
2		0.75nM	24h	11482548	4962285	0.434	2766614	0.241	13296210	8621795	0.648	11897280	1291850	0.109
3		0.75nM	48h	13051852	3831072	0.294	2420022	0.185	14833690	7910176	0.533	13186090	810880	0.061
4		3.16nM	24h	10811328	8060672	0.747	12537180	1.160	11690464	6930976	0.584	10905984	1044960	0.096
5	JJ012	-	48h	12489280	9765980	0.782	7453616	0.597	13473888	13890310	1.031	12969056	465675	0.036
6		1nM	24h	12092928	10152736	0.840	6856113	0.567	12078592	12696832	1.051	12047008	503904	0.042
7		1nM	48h	13875488	6316192	0.455	8905786	0.642	14527792	11517792	0.793	14266464	433088	0.030
8		3.16nM	24h	11088808	10595424	0.956	15400350	1.389	11009888	10458384	0.950	11877184	414240	0.035
9	SW1353	-	48h	12052928	6096755	0.506	4843509	0.402	12494944	19439420	1.556	13000832	1088885	0.084
10		0.75nM	24h	11908160	6222816	0.523	4745006	0.398	11803360	18424096	1.561	12299616	966816	0.079
11		0.75nM	48h	12639680	3767855	0.298	5751168	0.455	13562241	18015025	1.328	13506446	779030	0.058
12		3.16nM	24h	9036800	4023824	0.445	17138862	1.897	9039200	12107251	1.339	8971360	819217	0.091

	Romidepsin			α-Tubulin Intensity	Bak Intensity	Bak Ratio	Bax Intensity	Bax Ratio	α-Tubulin Intensity	Bcl-w Intensity	Bcl-w Ratio	α-Tubulin Intensity	Bid Intensity	Bid Ratio
1	CH2879	-	48h	12155060	3261885	0.268	1601950	0.132	10817079	317536	0.029	13328310	10061488	0.755
2		0.75nM	24h	10864920	3341996	0.308	1722032	0.158	10121154	568210	0.056	11651442	12095358	1.038
3		0.75nM	48h	12077590	3230667	0.267	1972740	0.163	11208882	537568	0.048	12775560	12918815	1.011
4		3.16nM	24h	10687606	3304980	0.309	1806732	0.169	9437536	194720	0.021	10295585	10458108	1.016
5	JJ012	-	48h	11733842	4597650	0.392	1735836	0.148	10683584	237230	0.022	12059664	9121010	0.756
6		1nM	24h	10946892	5129273	0.469	1800015	0.164	9509600	215104	0.023	11250030	9213303	0.819
7		1nM	48h	13042512	5497884	0.422	1639584	0.126	11531360	151680	0.013	13222656	8128080	0.615
8		3.16nM	24h	10824992	5977755	0.552	1797145	0.166	9791200	60352	0.006	10680448	7596995	0.711
9	SW1353	-	48h	12069216	5186020	0.430	1733375	0.144	10846240	422065	0.039	11611648	8477980	0.730
10		0.75nM	24h	12293482	5648318	0.459	1804584	0.147	10874496	322464	0.030	11306394	7786312	0.689
11		0.75nM	48h	13074190	6169912	0.472	1552304	0.119	11733376	108850	0.009	12506016	6636834	0.531
12		3.16nM	24h	9040944	5543901	0.613	1330560	0.147	7685566	41921	0.005	9309927	5004681	0.538

E

Whole Blots Figure S4

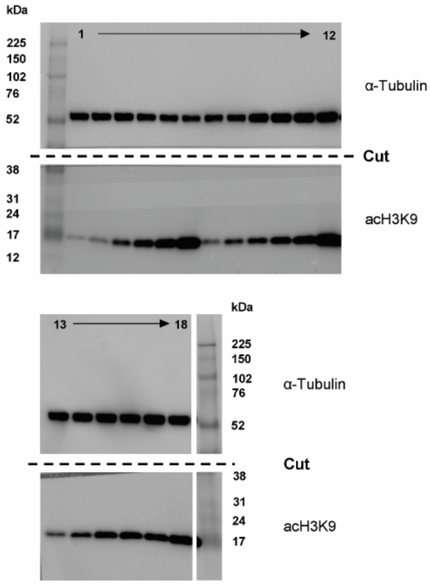

	Romidepsin			α-Tubulin Intensity	acH3K9 Intensity	acH3K9 Ratio
1	SW1353	-	24h	5681190	1141406	0.201
2		0.316nM	24h	6293720	2023880	0.322
3		0.562nM	24h	7219602	4425837	0.613
4		0.75nM	24h	5497460	7542658	1.372
5		1nM	24h	5394012	12472083	2.312
6		3.16nM	24h	5667012	23742900	4.190
7	JJ012	-	24h	7314696	1474932	0.202
8		0.562nM	24h	7137760	2016320	0.282
9		0.75nM	24h	9726912	2615535	0.269
10		1nM	24h	12159400	4001096	0.329
11		1.33nM	24h	13393968	5905614	0.441
12		3.16nM	24h	15982800	17000640	1.064
13	CH2879	-	24h	8652228	3824721	0.442
14		0.316nM	24h	9538933	5814512	0.610
15		0.562nM	24h	11149162	9342036	0.838
16		0.75nM	24h	10661217	10198095	0.957
17		1nM	24h	13297074	10523834	0.791
18		3.16nM	24h	12175605	14743066	1.211

Figure S7. Whole blots with densitometry readings from the performed western blots. Whole blots with densitometry readings from the western blots depicted in (**A**) Figure 4D, (**B**) Figure 5B, (**C**) Figure 6C, (**D**) Figure 6D, and (**E**) Figure S4.

Table S1. Characteristics of patient samples that were used for the DNA methylation array.

Sample ID	Tumor Location	Tumor Type	Grade	*IDH1* Mutation	*IDH2* Mutation	CIMP-Status
L19	Femur	CS	II	-	R172S	Positive
L186	Scapula	CS	II	R132C	-	Positive
L205	Humerus	EC	-	-	R172M	Positive
L223	Tibia	CS	II	R132C	-	Positive
L314	Radius	CS	ACT/I	R132H	-	Positive
L533	Humerus	CS	ACT/I	R132S	-	Negative
L646	Unknown	CS	II	R132C	-	Positive
L738	Unknown	CS	ACT/I	R132S	-	Positive
L855	Digit	CS	II	R132G	-	Positive
L869	Tibia	CS	II	R132C	-	Positive
L1326	Axilla	CS	III	R132C	-	Positive
L1491	Ulna	EC	-	R132H	-	Positive
L1536	Unknown	CS	II	R132G	-	Positive
L1539	Femur	CS	ACT/I	R132L	-	Positive
L1769	Femur	CS	ACT/I	R132H	-	Negative
L1829	Tibia	EC	-	R132C	-	Positive
L1993	Unknown	CS	III	R132C	-	Positive
L2088	Femur	CS	III	R132C	-	Positive
L2814	Femur	CS	II	R132C	-	Positive
L3529	Femur	CS	ACT/I	R132S	-	Positive

EC: enchondroma, ACT: atypical cartilaginous tumor, CS: chondrosarcoma.

Table S2. Significantly differentially methylated genes in benign/low-grade and high-grade cartilage tumors.

**Strongly Methylated in Benign/Low-Grade Tumors (*n* = 89)**		
**Gene**	***p*-Value**	**Difference in β-Value**
ZFAND2A	1.82×10^{-6}	-0.074
DUSP14	3.37×10^{-6}	-0.105
TNFRSF25	1.05×10^{-5}	-0.334
TCEA2	1.82×10^{-5}	-0.068
DIXDC1	2.12×10^{-5}	-0.067
PHC3	3.68×10^{-5}	-0.053
DUS1L	5.77×10^{-5}	-0.086
MT2A	6.62×10^{-5}	-0.135
C9orf69	6.98×10^{-5}	-0.038
VPS13D	7.60×10^{-5}	-0.045
VEGFA	7.90×10^{-5}	-0.096
MRPL11	9.18×10^{-5}	-0.050
SERPINH1	1.00×10^{-4}	-0.052
C3orf21	1.05×10^{-4}	-0.046
RER1	1.31×10^{-4}	-0.066
MORN1	1.31×10^{-4}	-0.066
B4GALT4	1.39×10^{-4}	-0.019
KCTD11	1.42×10^{-4}	-0.060
C5orf27	1.43×10^{-4}	-0.065
ZMYND8	2.14×10^{-4}	-0.061
WHAMM	2.41×10^{-4}	-0.061
C14orf79	2.44×10^{-4}	-0.016
STK3	2.75×10^{-4}	-0.052
TRIM4	2.91×10^{-4}	-0.035
C18orf45	2.95×10^{-4}	-0.080
FRMD4A	2.99×10^{-4}	-0.213
RNU6ATAC	3.63×10^{-4}	-0.063
RIN1	3.72×10^{-4}	-0.082
KLHL21	3.78×10^{-4}	-0.044
PNPLA2	3.84×10^{-4}	-0.017
C19orf61	5.11×10^{-4}	-0.033
PSMD9	5.45×10^{-4}	-0.024
ARFGAP1	5.51×10^{-4}	-0.021
TMEM121	6.04×10^{-4}	-0.067
KAZALD1	6.76×10^{-4}	-0.041
MINPP1	8.02×10^{-4}	-0.060
TRAF4	8.87×10^{-4}	-0.004
SNRPD1	9.02×10^{-4}	-0.031
GADD45B	9.68×10^{-4}	-0.031
DNAJB13	1.00×10^{-3}	-0.381
SPATA6	1.00×10^{-3}	-0.018
RBM28	1.09×10^{-3}	-0.010
EXOG	1.11×10^{-3}	-0.049
SOCS3	1.11×10^{-3}	-0.111
ORAOV1	1.21×10^{-3}	-0.021
HLCS	1.21×10^{-3}	-0.039
ATP8B4	1.22×10^{-3}	-0.290
GAPDH	1.29×10^{-3}	-0.025
GINS4	1.34×10^{-3}	-0.031
PPM1G	1.35×10^{-3}	-0.039
PLCL2	1.40×10^{-3}	-0.218
SNX21	1.42×10^{-3}	-0.022
GPS2	1.42×10^{-3}	-0.062
ANKRD46	1.45×10^{-3}	-0.016
CCNH	1.47×10^{-3}	-0.016
PATL1	1.69×10^{-3}	-0.034
C3orf67	1.72×10^{-3}	-0.031
ITCH	1.75×10^{-3}	-0.020
ASXL2	1.80×10^{-3}	-0.001
PHKG2	1.87×10^{-3}	-0.017
GARS	1.91×10^{-3}	-0.034
MIR548H4	1.95×10^{-3}	-0.021
GLCE	1.95×10^{-3}	-0.021
ADRM1	1.97×10^{-3}	-0.047
BPHL	2.01×10^{-3}	-0.024
DKFZP686I15217	2.14×10^{-3}	-0.005

AGFG1	2.19×10^{-3}	-0.063
FAM188A	2.21×10^{-3}	-0.044
DUSP19	2.22×10^{-3}	-0.008
OGG1	2.24×10^{-3}	-0.025
BBS2	2.26×10^{-3}	-0.021
USP22	2.26×10^{-3}	-0.011
TM2D3	2.27×10^{-3}	-0.061
DCTN4	2.28×10^{-3}	-0.041
ABHD14B	2.33×10^{-3}	-0.018
ABHD14A	2.33×10^{-3}	-0.018
RPL27A	2.42×10^{-3}	-0.009
TSSK6	2.50×10^{-3}	-0.071
NDUFA13	2.50×10^{-3}	-0.071
SENP5	2.58×10^{-3}	-0.013
MAMSTR	2.71×10^{-3}	-0.121
FAM91A1	2.80×10^{-3}	-0.018
PPP2CB	2.94×10^{-3}	-0.076
FNDC8	3.11×10^{-3}	-0.164
ZNF34	3.14×10^{-3}	-0.039
ZFP36	3.26×10^{-3}	-0.022
SLC22A15	3.41×10^{-3}	-0.215
CENPH	3.41×10^{-3}	-0.040
DGCR6	3.47×10^{-3}	-0.041
**Strongly Methylated in High-Grade Tumors (n = 592)**		
**Gene**	**p-Value**	**Difference in β-Value**
CEBPA	1.64×10^{-12}	0.311
LBX2	1.80×10^{-10}	0.488
SLC17A9	4.60×10^{-9}	0.455
C9orf167	6.79×10^{-9}	0.459
KCTD1	7.01×10^{-9}	0.159
CDCA7L	2.17×10^{-8}	0.367
SPRED3	2.27×10^{-8}	0.360
PROCA1	2.33×10^{-8}	0.319
AP1S3	2.93×10^{-8}	0.135
NRBP1	3.09×10^{-8}	0.054
RNF122	7.08×10^{-8}	0.057
CDKN2BAS	9.92×10^{-8}	0.087
ITGB7	1.02×10^{-7}	0.274
CHD5	1.26×10^{-7}	0.308
REC8	1.85×10^{-7}	0.273
ATG9B	2.27×10^{-7}	0.208
NOS3	2.27×10^{-7}	0.208
PDE4C	2.41×10^{-7}	0.138
NCRNA00085	2.69×10^{-7}	0.385
ZNF771	3.15×10^{-7}	0.161
P2RY1	3.92×10^{-7}	0.336
PTGER4	4.24×10^{-7}	0.356
MAL	5.20×10^{-7}	0.140
STX1B	6.11×10^{-7}	0.215
CRIP1	7.05×10^{-7}	0.333
EMILIN2	7.39×10^{-7}	0.181
SARM1	7.62×10^{-7}	0.191
PIK3AP1	8.33×10^{-7}	0.118
ARNTL2	8.69×10^{-7}	0.108
SYNGR3	8.97×10^{-7}	0.332
SEL1L3	1.29×10^{-6}	0.275
FOXP4	1.44×10^{-6}	0.271
NFATC2	1.49×10^{-6}	0.221
FUZ	1.59×10^{-6}	0.121
CYBA	1.62×10^{-6}	0.447
RINL	1.65×10^{-6}	0.244
PCDHGC5	1.74×10^{-6}	0.271
PCDHGC4	1.74×10^{-6}	0.271
CACNA1C	1.75×10^{-6}	0.319
ADAM8	1.81×10^{-6}	0.391
VSIG10L	1.97×10^{-6}	0.153
BZRAP1	2.06×10^{-6}	0.234
IGF1R	2.07×10^{-6}	0.188
GMPPA	2.13×10^{-6}	0.073
TBC1D10C	2.22×10^{-6}	0.130

C8orf73	2.43×10^{-6}	0.341
ABCC1	2.47×10^{-6}	0.043
ACP5	2.51×10^{-6}	0.229
HRAS	2.56×10^{-6}	0.166
B4GALT6	3.13×10^{-6}	0.183
TRAF5	3.22×10^{-6}	0.093
AFF1	3.45×10^{-6}	0.073
EPC1	3.52×10^{-6}	0.129
MGA	3.69×10^{-6}	0.279
SH2D3A	4.24×10^{-6}	0.258
RREB1	4.41×10^{-6}	0.128
HIST1H3J	4.70×10^{-6}	0.446
ICAM5	5.09×10^{-6}	0.369
PCDHGA4	5.17×10^{-6}	0.083
PCDHGA12	5.17×10^{-6}	0.083
PCDHGA11	5.17×10^{-6}	0.083
PCDHGA9	5.17×10^{-6}	0.083
PCDHGA1	5.17×10^{-6}	0.083
PCDHGB1	5.17×10^{-6}	0.083
PCDHGC3	5.17×10^{-6}	0.083
PCDHGB6	5.17×10^{-6}	0.083
PCDHGB3	5.17×10^{-6}	0.083
PCDHGB7	5.17×10^{-6}	0.083
PCDHGA6	5.17×10^{-6}	0.083
PCDHGA8	5.17×10^{-6}	0.083
PCDHGA10	5.17×10^{-6}	0.083
PCDHGA5	5.17×10^{-6}	0.083
PCDHGB4	5.17×10^{-6}	0.083
PCDHGA3	5.17×10^{-6}	0.083
PCDHGA2	5.17×10^{-6}	0.083
PCDHGA7	5.17×10^{-6}	0.083
PCDHGB2	5.17×10^{-6}	0.083
PCDHGB5	5.17×10^{-6}	0.083
ENC1	5.43×10^{-6}	0.162
ST6GAL1	5.82×10^{-6}	0.241
ALDH5A1	6.08×10^{-6}	0.182
PHF11	6.09×10^{-6}	0.201
ITPKA	6.13×10^{-6}	0.425
STAT4	6.42×10^{-6}	0.319
IFI30	6.45×10^{-6}	0.163
ADRBK1	6.48×10^{-6}	0.134
DMC1	6.71×10^{-6}	0.264
HIST1H4D	7.68×10^{-6}	0.273
ADORA2A	8.84×10^{-6}	0.161
ABI3	9.09×10^{-6}	0.526
GNGT2	9.09×10^{-6}	0.526
DHH	9.11×10^{-6}	0.349
DNAH3	9.27×10^{-6}	0.139
TMEM159	9.27×10^{-6}	0.139
FAM110A	9.74×10^{-6}	0.156
LMTK3	1.10×10^{-5}	0.111
IL23A	1.16×10^{-5}	0.256
CORO6	1.19×10^{-5}	0.203
LFNG	1.19×10^{-5}	0.102
TBC1D1	1.25×10^{-5}	0.072
TNFRSF1B	1.36×10^{-5}	0.192
TYMP	1.47×10^{-5}	0.153
LIMD2	1.48×10^{-5}	0.132
IKZF3	1.52×10^{-5}	0.221
ZNF808	1.56×10^{-5}	0.514
C17orf46	1.56×10^{-5}	0.355
NTNG2	1.57×10^{-5}	0.161
IL15RA	1.62×10^{-5}	0.126
SLCO5A1	1.63×10^{-5}	0.175
CD74	1.64×10^{-5}	0.263
KIAA1522	1.71×10^{-5}	0.106
MGC29506	1.81×10^{-5}	0.129
CACNA2D2	1.92×10^{-5}	0.290
S1PR4	2.06×10^{-5}	0.150
KIAA0562	2.11×10^{-5}	0.063

DFFB	2.23×10^{-5}	0.059
FGR	2.27×10^{-5}	0.161
GOLM1	2.28×10^{-5}	0.354
TM7SF2	2.28×10^{-5}	0.331
FCHO1	2.32×10^{-5}	0.148
WNT1	2.35×10^{-5}	0.269
BATF3	2.37×10^{-5}	0.145
MKNK2	2.40×10^{-5}	0.213
RRBP1	2.43×10^{-5}	0.047
KIAA1274	2.44×10^{-5}	0.126
NHLRC4	2.47×10^{-5}	0.081
FAM107B	2.47×10^{-5}	0.171
HTATIP2	2.49×10^{-5}	0.323
CYTH2	2.54×10^{-5}	0.095
HHEX	2.56×10^{-5}	0.119
LIPE	2.71×10^{-5}	0.228
INPP5D	2.73×10^{-5}	0.154
LRRC8C	2.89×10^{-5}	0.063
LOC606724	2.89×10^{-5}	0.116
RAB24	2.99×10^{-5}	0.038
GADD45G	3.00×10^{-5}	0.302
C9orf66	3.00×10^{-5}	0.268
DOCK8	3.00×10^{-5}	0.268
ZBTB44	3.08×10^{-5}	0.049
OSM	3.08×10^{-5}	0.132
PRR5L	3.16×10^{-5}	0.105
C8orf51	3.18×10^{-5}	0.077
RHPN1	3.18×10^{-5}	0.077
ZNF296	3.43×10^{-5}	0.281
KRTCAP3	3.43×10^{-5}	0.289
FAM78A	3.74×10^{-5}	0.222
SLC9A3R1	3.82×10^{-5}	0.111
SERPINB9	3.90×10^{-5}	0.325
HMHA1	4.02×10^{-5}	0.134
DNMT3A	4.04×10^{-5}	0.146
CARHSP1	4.41×10^{-5}	0.048
SLC29A2	4.43×10^{-5}	0.089
CFLAR	4.58×10^{-5}	0.227
GDI2	4.63×10^{-5}	0.029
HDAC3	4.78×10^{-5}	0.115
RELL2	4.78×10^{-5}	0.115
FRAT1	4.79×10^{-5}	0.071
IER3	5.54×10^{-5}	0.258
QRFP	5.64×10^{-5}	0.117
ONECUT1	5.66×10^{-5}	0.237
PYCARD	5.70×10^{-5}	0.264
SH2B3	5.70×10^{-5}	0.110
LPAR2	5.79×10^{-5}	0.329
TK2	5.88×10^{-5}	0.062
C1orf38	6.05×10^{-5}	0.120
LRRC20	6.05×10^{-5}	0.089
ATP2B4	6.18×10^{-5}	0.139
LOC146880	6.20×10^{-5}	0.380
BMP8B	6.39×10^{-5}	0.179
ABCA7	6.55×10^{-5}	0.097
LOC100133991	6.70×10^{-5}	0.199
CDKN2A	6.72×10^{-5}	0.125
FERMT3	6.90×10^{-5}	0.136
RAB3D	6.92×10^{-5}	0.327
CCDC88B	7.08×10^{-5}	0.048
C17orf62	7.28×10^{-5}	0.094
LTB4R2	7.46×10^{-5}	0.211
LTB4R	7.46×10^{-5}	0.211
CIDEB	7.46×10^{-5}	0.211
PTEN	7.50×10^{-5}	0.333
KILLIN	7.50×10^{-5}	0.333
EPHA4	7.54×10^{-5}	0.079
UBE2Q2	7.67×10^{-5}	0.261
SNN	7.67×10^{-5}	0.065
HCST	8.21×10^{-5}	0.382

UNKL	8.51×10^{-5}	0.088
ABCG1	8.57×10^{-5}	0.145
NTN1	8.59×10^{-5}	0.334
CHST12	8.74×10^{-5}	0.058
NRGN	8.87×10^{-5}	0.339
ANKRD23	9.15×10^{-5}	0.265
MFNG	9.17×10^{-5}	0.126
PRAGMIN	9.38×10^{-5}	0.238
CYP2U1	9.53×10^{-5}	0.098
HMGA1	9.80×10^{-5}	0.097
MYD88	9.84×10^{-5}	0.285
FAM102B	9.84×10^{-5}	0.112
BTBD11	9.88×10^{-5}	0.123
EMP3	1.01×10^{-4}	0.162
TNFRSF10A	1.05×10^{-4}	0.219
ANKRD28	1.11×10^{-4}	0.105
STK38	1.13×10^{-4}	0.025
MAP4K1	1.17×10^{-4}	0.073
EIF3K	1.17×10^{-4}	0.073
SNED1	1.17×10^{-4}	0.095
ISL2	1.17×10^{-4}	0.236
ALOX5AP	1.21×10^{-4}	0.175
MST1R	1.22×10^{-4}	0.125
HSBP1L1	1.24×10^{-4}	0.145
NUP210	1.29×10^{-4}	0.105
SNX32	1.30×10^{-4}	0.056
ACAA1	1.31×10^{-4}	0.228
CDC42BPG	1.32×10^{-4}	0.075
PIK3CD	1.33×10^{-4}	0.141
IL16	1.35×10^{-4}	0.058
RLTPR	1.39×10^{-4}	0.153
KCNAB2	1.40×10^{-4}	0.119
ETS2	1.43×10^{-4}	0.107
C9orf78	1.44×10^{-4}	0.081
USP20	1.44×10^{-4}	0.081
ANKRD34A	1.45×10^{-4}	0.028
RAB19	1.48×10^{-4}	0.330
KCNQ4	1.49×10^{-4}	0.329
SYNJ2	1.54×10^{-4}	0.060
DISP2	1.65×10^{-4}	0.057
GYG1	1.67×10^{-4}	0.017
ACP1	1.68×10^{-4}	0.058
SH3YL1	1.68×10^{-4}	0.058
LRRC37A3	1.77×10^{-4}	0.120
ALOX5	1.78×10^{-4}	0.236
B3GNT2	1.79×10^{-4}	0.175
GPR160	1.80×10^{-4}	0.060
TRAF1	1.81×10^{-4}	0.431
LYL1	1.82×10^{-4}	0.285
C14orf169	1.85×10^{-4}	0.190
HEATR4	1.85×10^{-4}	0.190
ADAMTSL5	1.87×10^{-4}	0.362
TRAPPC5	1.88×10^{-4}	0.083
RNF187	1.95×10^{-4}	0.046
ANKLE1	1.95×10^{-4}	0.240
NT5C3	1.95×10^{-4}	0.025
KLHDC5	1.96×10^{-4}	0.109
TP53TG5	2.00×10^{-4}	0.489
GJD3	2.10×10^{-4}	0.118
COTL1	2.13×10^{-4}	0.030
AXIN1	2.22×10^{-4}	0.057
HCG4P6	2.28×10^{-4}	0.299
ICAM1	2.30×10^{-4}	0.218
ZFAND2B	2.32×10^{-4}	0.024
PRKAG2	2.35×10^{-4}	0.101
FABP5	2.38×10^{-4}	0.302
EML5	2.38×10^{-4}	0.162
IL4I1	2.46×10^{-4}	0.065
NFIL3	2.47×10^{-4}	0.103
TMC4	2.53×10^{-4}	0.144

YPEL1	2.54×10^{-4}	0.062
LRPAP1	2.59×10^{-4}	0.061
EFCAB4B	2.60×10^{-4}	0.202
SLFN13	2.60×10^{-4}	0.275
NAGK	2.60×10^{-4}	0.042
GPR68	2.64×10^{-4}	0.141
RASSF2	2.67×10^{-4}	0.146
MTA2	2.67×10^{-4}	0.077
THSD1P	2.68×10^{-4}	0.131
CBX4	2.75×10^{-4}	0.042
RAB11FIP4	2.79×10^{-4}	0.234
TOX	2.79×10^{-4}	0.159
TTBK1	2.80×10^{-4}	0.209
MAPRE2	2.81×10^{-4}	0.161
LOC220930	2.85×10^{-4}	0.030
POU6F1	2.86×10^{-4}	0.032
C16orf54	2.87×10^{-4}	0.144
LYPD3	2.89×10^{-4}	0.274
CLSTN3	2.94×10^{-4}	0.398
CASP6	2.97×10^{-4}	0.021
MGAT1	2.98×10^{-4}	0.059
USP34	2.99×10^{-4}	0.079
MFSD2A	3.00×10^{-4}	0.294
EPSTI1	3.01×10^{-4}	0.290
SLC2A9	3.02×10^{-4}	0.157
IQGAP2	3.03×10^{-4}	0.163
TXNRD1	3.04×10^{-4}	0.200
DDX39	3.06×10^{-4}	0.274
CSK	3.06×10^{-4}	0.051
GMEB1	3.08×10^{-4}	0.055
ADD1	3.08×10^{-4}	0.047
FLI1	3.09×10^{-4}	0.170
ZMYM2	3.22×10^{-4}	0.086
RAB37	3.22×10^{-4}	0.236
PARP12	3.26×10^{-4}	0.125
PGS1	3.26×10^{-4}	0.066
ZC3HAV1	3.31×10^{-4}	0.056
ADCY4	3.32×10^{-4}	0.216
PPP1R15A	3.34×10^{-4}	0.036
ZFP90	3.37×10^{-4}	0.082
PRSS27	3.45×10^{-4}	0.165
TEC	3.50×10^{-4}	0.155
NMNAT3	3.51×10^{-4}	0.202
MEST	3.53×10^{-4}	0.103
HERC5	3.57×10^{-4}	0.070
CORO1A	3.61×10^{-4}	0.052
AMZ1	3.62×10^{-4}	0.248
RGS16	3.70×10^{-4}	0.261
VAV1	3.70×10^{-4}	0.171
LDLRAD2	3.73×10^{-4}	0.151
RNF44	3.80×10^{-4}	0.036
ZNF254	3.88×10^{-4}	0.470
FAM55C	3.89×10^{-4}	0.152
CD38	3.95×10^{-4}	0.140
CCDC61	4.02×10^{-4}	0.049
EML3	4.06×10^{-4}	0.053
CLIP2	4.07×10^{-4}	0.021
DSCR9	4.10×10^{-4}	0.151
HIST3H2BB	4.16×10^{-4}	0.296
C21orf122	4.20×10^{-4}	0.023
HLA-L	4.21×10^{-4}	0.085
FLYWCH2	4.31×10^{-4}	0.025
PTGER2	4.32×10^{-4}	0.303
SUSD1	4.37×10^{-4}	0.170
KLHDC7B	4.41×10^{-4}	0.180
SEMA7A	4.48×10^{-4}	0.187
FLOT1	4.52×10^{-4}	0.101
ZFPM1	4.61×10^{-4}	0.347
LOC651250	4.67×10^{-4}	0.161
ZEB1	4.74×10^{-4}	0.025

HPCAL1	4.75×10^{-4}	0.090
PTPRCAP	4.81×10^{-4}	0.092
QPCT	4.83×10^{-4}	0.279
RIPK3	4.87×10^{-4}	0.209
TRAPPC9	4.97×10^{-4}	0.056
SHF	4.97×10^{-4}	0.145
SFMBT1	4.99×10^{-4}	0.231
LOC100130557	4.99×10^{-4}	0.056
NUP153	5.03×10^{-4}	0.030
CCNI2	5.05×10^{-4}	0.123
TRIM59	5.15×10^{-4}	0.061
RBP5	5.17×10^{-4}	0.344
WIPF1	5.28×10^{-4}	0.049
BAMBI	5.43×10^{-4}	0.117
LINGO3	5.51×10^{-4}	0.414
DGAT2	5.54×10^{-4}	0.114
KCNIP2	5.55×10^{-4}	0.270
PAOX	5.56×10^{-4}	0.105
TMEM154	5.58×10^{-4}	0.101
LGALS9	5.58×10^{-4}	0.338
SIPA1	5.62×10^{-4}	0.222
CD72	5.64×10^{-4}	0.247
ZDHHC24	5.72×10^{-4}	0.136
ZEB2	5.95×10^{-4}	0.401
SNORA16A	5.97×10^{-4}	0.056
SNHG12	5.97×10^{-4}	0.056
POU3F2	5.98×10^{-4}	0.041
PATZ1	6.13×10^{-4}	0.100
HIST3H2A	6.15×10^{-4}	0.244
C11orf91	6.20×10^{-4}	0.143
CENPV	6.28×10^{-4}	0.141
CBY3	6.32×10^{-4}	0.094
GRK5	6.33×10^{-4}	0.041
TSPAN14	6.43×10^{-4}	0.029
MARCH3	6.52×10^{-4}	0.050
RASL11B	6.54×10^{-4}	0.162
CAMK1D	6.64×10^{-4}	0.113
TPM1	6.64×10^{-4}	0.227
CDKL2	6.78×10^{-4}	0.263
SNORD65	6.96×10^{-4}	0.112
MCF2L2	7.02×10^{-4}	0.080
BCAT1	7.26×10^{-4}	0.122
sep-01	7.49×10^{-4}	0.042
FLOT2	7.69×10^{-4}	0.030
TNPO2	7.69×10^{-4}	0.108
C5orf39	7.74×10^{-4}	0.304
RFC4	7.78×10^{-4}	0.009
MTRF1	8.00×10^{-4}	0.012
SPPL3	8.05×10^{-4}	0.065
PKN1	8.05×10^{-4}	0.054
ACTN3	8.10×10^{-4}	0.117
C9orf98	8.19×10^{-4}	0.132
NKX3-1	8.21×10^{-4}	0.106
CUL9	8.33×10^{-4}	0.082
NAGLU	8.49×10^{-4}	0.034
ANKRD53	8.51×10^{-4}	0.188
HEATR6	8.62×10^{-4}	0.055
ITGB2	8.72×10^{-4}	0.072
RPP25	8.82×10^{-4}	0.148
ADARB1	8.92×10^{-4}	0.018
TUBG2	8.97×10^{-4}	0.033
ZWILCH	9.01×10^{-4}	0.030
RPL4	9.01×10^{-4}	0.030
HLA-E	9.05×10^{-4}	0.064
PHLDA1	9.12×10^{-4}	0.206
SLC39A14	9.23×10^{-4}	0.077
ICAM3	9.25×10^{-4}	0.091
HPSE2	9.27×10^{-4}	0.092
SYNPO	9.28×10^{-4}	0.387
LY75	9.33×10^{-4}	0.218

KDM2B	9.33×10^{-4}	0.116
POMP	9.33×10^{-4}	0.029
ATP2A3	9.35×10^{-4}	0.099
BIRC3	9.41×10^{-4}	0.335
SORBS3	9.42×10^{-4}	0.237
RGS3	9.49×10^{-4}	0.155
WDR8	9.68×10^{-4}	0.059
CMC1	9.88×10^{-4}	0.020
KLF13	9.93×10^{-4}	0.106
C18orf1	1.00×10^{-3}	0.286
CTSZ	1.01×10^{-3}	0.047
FBXL22	1.06×10^{-3}	0.036
CCDC48	1.06×10^{-3}	0.245
PLAUR	1.10×10^{-3}	0.106
ATP11B	1.10×10^{-3}	0.063
ZC3HAV1L	1.11×10^{-3}	0.177
C19orf76	1.13×10^{-3}	0.259
BCL2	1.13×10^{-3}	0.140
VOPP1	1.14×10^{-3}	0.096
GPR3	1.16×10^{-3}	0.102
DNAH10	1.16×10^{-3}	0.347
MAP7D1	1.17×10^{-3}	0.069
TRIM2	1.18×10^{-3}	0.165
C14orf43	1.19×10^{-3}	0.025
UNC84B	1.19×10^{-3}	0.057
C1orf88	1.19×10^{-3}	0.102
SGEF	1.21×10^{-3}	0.120
AGMAT	1.21×10^{-3}	0.160
CD55	1.21×10^{-3}	0.064
C10orf18	1.23×10^{-3}	0.093
SGK1	1.26×10^{-3}	0.142
SRCIN1	1.27×10^{-3}	0.116
EPS8	1.27×10^{-3}	0.345
LRCH4	1.31×10^{-3}	0.037
SQRDL	1.32×10^{-3}	0.162
CDK5R1	1.35×10^{-3}	0.030
MICB	1.35×10^{-3}	0.049
HMGCL	1.37×10^{-3}	0.061
HIVEP2	1.37×10^{-3}	0.040
ZNF783	1.40×10^{-3}	0.137
NFKBIZ	1.40×10^{-3}	0.224
ITGA4	1.41×10^{-3}	0.205
NRARP	1.42×10^{-3}	0.143
TGFB1I1	1.42×10^{-3}	0.098
CPM	1.43×10^{-3}	0.222
FAM43A	1.43×10^{-3}	0.129
SORL1	1.43×10^{-3}	0.122
HDAC7	1.44×10^{-3}	0.138
TMEM134	1.46×10^{-3}	0.076
ARHGDIB	1.46×10^{-3}	0.108
RGS9BP	1.46×10^{-3}	0.050
DKFZp686O24166	1.48×10^{-3}	0.290
LARP1	1.51×10^{-3}	0.115
MAP4K2	1.52×10^{-3}	0.053
SPRY1	1.54×10^{-3}	0.181
SBK1	1.55×10^{-3}	0.234
ABLIM1	1.56×10^{-3}	0.194
RALGAPA2	1.57×10^{-3}	0.059
BANP	1.58×10^{-3}	0.024
DKFZp761E198	1.58×10^{-3}	0.047
SLC38A2	1.60×10^{-3}	0.048
ESRP2	1.62×10^{-3}	0.109
ANP32A	1.65×10^{-3}	0.061
ITPR3	1.65×10^{-3}	0.055
SLFN12L	1.66×10^{-3}	0.300
TMPRSS8	1.67×10^{-3}	0.221
DGKE	1.69×10^{-3}	0.099
ARPC1B	1.72×10^{-3}	0.181
CORIN	1.72×10^{-3}	0.112
SLC38A10	1.74×10^{-3}	0.095

MYO15A	1.74 × 10⁻³	0.171
MTMR9L	1.76 × 10⁻³	0.116
LRMP	1.77 × 10⁻³	0.098
SIGIRR	1.78 × 10⁻³	0.114
BEST4	1.78 × 10⁻³	0.218
PVT1	1.79 × 10⁻³	0.026
NRTN	1.79 × 10⁻³	0.251
PLEKHG3	1.80 × 10⁻³	0.216
NFIC	1.82 × 10⁻³	0.121
KCNMB4	1.86 × 10⁻³	0.106
TTC23L	1.87 × 10⁻³	0.130
LSP1	1.88 × 10⁻³	0.128
PNPLA7	1.90 × 10⁻³	0.070
LYSMD2	1.90 × 10⁻³	0.118
CES8	1.91 × 10⁻³	0.156
PARP10	1.93 × 10⁻³	0.042
GALK2	1.94 × 10⁻³	0.038
LRRC14	1.95 × 10⁻³	0.091
RASSF5	1.95 × 10⁻³	0.068
VILL	1.95 × 10⁻³	0.211
CYB5A	1.96 × 10⁻³	0.308
SLMO2	1.97 × 10⁻³	0.045
NEDD9	1.99 × 10⁻³	0.036
MIR142	1.99 × 10⁻³	0.121
LOC100268168	1.99 × 10⁻³	0.070
RPL26L1	1.99 × 10⁻³	0.070
FOXN3	1.99 × 10⁻³	0.082
IL12RB2	2.00 × 10⁻³	0.266
FXYD5	2.02 × 10⁻³	0.109
FHIT	2.05 × 10⁻³	0.107
TNK1	2.05 × 10⁻³	0.099
ZNF710	2.05 × 10⁻³	0.035
NT5DC3	2.05 × 10⁻³	0.169
HPDL	2.06 × 10⁻³	0.209
UBE2E2	2.08 × 10⁻³	0.180
USP45	2.08 × 10⁻³	0.005
SLC25A30	2.09 × 10⁻³	0.028
C16orf86	2.10 × 10⁻³	0.142
C16orf48	2.10 × 10⁻³	0.142
ZBTB12	2.11 × 10⁻³	0.036
STX12	2.11 × 10⁻³	0.031
GFI1	2.12 × 10⁻³	0.141
TMC6	2.13 × 10⁻³	0.095
CD300A	2.14 × 10⁻³	0.163
FLJ36777	2.14 × 10⁻³	0.240
DLG4	2.14 × 10⁻³	0.096
ZNF321	2.14 × 10⁻³	0.017
S100A6	2.15 × 10⁻³	0.201
SIAH2	2.15 × 10⁻³	0.034
TTC17	2.20 × 10⁻³	0.021
RASGRP1	2.20 × 10⁻³	0.074
PCNX	2.20 × 10⁻³	0.010
ABCA1	2.22 × 10⁻³	0.187
IVNS1ABP	2.22 × 10⁻³	0.066
C5orf24	2.24 × 10⁻³	0.025
DNM1	2.24 × 10⁻³	0.060
UHRF1	2.25 × 10⁻³	0.072
PTPN7	2.26 × 10⁻³	0.087
B3GALT4	2.28 × 10⁻³	0.128
TAF8	2.28 × 10⁻³	0.014
LATS1	2.30 × 10⁻³	0.008
NINJ2	2.30 × 10⁻³	0.094
LASP1	2.30 × 10⁻³	0.025
C2CD2L	2.31 × 10⁻³	0.032
NAPRT1	2.31 × 10⁻³	0.245
ZNF763	2.31 × 10⁻³	0.259
EVL	2.35 × 10⁻³	0.051
PER2	2.39 × 10⁻³	0.083
VIPR1	2.40 × 10⁻³	0.218
TSPYL5	2.40 × 10⁻³	0.174

HUS1	2.42×10^{-3}	0.126
RAC2	2.42×10^{-3}	0.146
RPS6KL1	2.43×10^{-3}	0.161
CELSR3	2.44×10^{-3}	0.162
COMMD10	2.45×10^{-3}	0.017
LRRC34	2.59×10^{-3}	0.241
AIG1	2.60×10^{-3}	0.006
AES	2.62×10^{-3}	0.204
CORO1B	2.63×10^{-3}	0.018
UBA2	2.64×10^{-3}	0.029
RRN3P1	2.65×10^{-3}	0.251
PDE6B	2.69×10^{-3}	0.061
C14orf182	2.69×10^{-3}	0.211
PLAU	2.70×10^{-3}	0.198
C10orf55	2.70×10^{-3}	0.198
APBB1IP	2.71×10^{-3}	0.225
WHSC1L1	2.72×10^{-3}	0.303
H2AFY	2.72×10^{-3}	0.059
AKAP1	2.72×10^{-3}	0.032
SLC38A1	2.75×10^{-3}	0.252
ATP1A3	2.75×10^{-3}	0.125
NR1I2	2.77×10^{-3}	0.196
COL23A1	2.82×10^{-3}	0.070
PCGF5	2.82×10^{-3}	0.083
C9orf142	2.86×10^{-3}	0.101
KIAA1324	2.86×10^{-3}	0.075
C1orf194	2.86×10^{-3}	0.075
CNDP2	2.89×10^{-3}	0.030
IRF4	2.94×10^{-3}	0.082
PRR5	2.96×10^{-3}	0.056
PRR5-ARHGAP8	2.96×10^{-3}	0.056
TXNRD2	2.97×10^{-3}	0.108
NIPAL1	2.99×10^{-3}	0.032
TBL1XR1	2.99×10^{-3}	0.023
TMC8	3.01×10^{-3}	0.093
C1orf59	3.04×10^{-3}	0.183
ZNF620	3.04×10^{-3}	0.045
FIP1L1	3.09×10^{-3}	0.030
DPP4	3.11×10^{-3}	0.143
DAPP1	3.15×10^{-3}	0.092
ZDHHC23	3.15×10^{-3}	0.041
ZBTB22	3.19×10^{-3}	0.042
UCP2	3.20×10^{-3}	0.223
PRDM8	3.21×10^{-3}	0.078
EZR	3.21×10^{-3}	0.087
RHOF	3.22×10^{-3}	0.083
ATP6V0C	3.22×10^{-3}	0.085
GPR137B	3.23×10^{-3}	0.051
CSRP1	3.28×10^{-3}	0.043
SPRED1	3.30×10^{-3}	0.129
BCL3	3.31×10^{-3}	0.046
C5orf56	3.37×10^{-3}	0.041
HIST1H3G	3.40×10^{-3}	0.244
GRIN2D	3.42×10^{-3}	0.130
CLIC5	3.44×10^{-3}	0.066
CTSC	3.45×10^{-3}	0.114
HLA-F	3.46×10^{-3}	0.110

Difference in β-value: high-grade β-value minus benign/low-grade β-value.

Table S3. Detailed list of all compounds included in the epigenetics compound library (L1900, Selleckchem).

Compound Class	Number	Product Name	Specific Targets
Aurora Kinases	1	Alisertib	Aurora A
	2	Aurora A Inhibitor I	Aurora A
	3	MK-5108	Aurora A
	4	Danusertib	Aurora A, Abl, RET, TrkA, FGFR1, Aurora C, Aurora B
	5	MLN8054	Aurora A, Aurora B
	6	CCT129202	Aurora A, Aurora B, Aurora C
	7	ZM 447439	Aurora A, Aurora B, LCK, Src, MEK1
	8	CYC116	Aurora A, Aurora B, VEGFR2, FLT3, CDK2, CDK9, p70 S6K
	9	CCT137690	Aurora A, Aurora C, Aurora B
	10	PHA-680632	Aurora A, Aurora C, Aurora B, FGFR1, PLK1, FLT3, VEGFR3, VEGFR2, LCK
	11	Tozasertib	Aurora A, Aurora C, Aurora B, FLT3, Bcr-Abl
	12	Barasertib	Aurora B
	13	Hesperadin	Aurora B
	14	SNS-314 Mesylate	Aurora C, Aurora A, Aurora B
	15	AMG-900	Aurora C, Aurora B, Aurora A, p38α
Bromodomain and Extra-Terminal Motif Proteins	16	RVX-208	BD2
	17	I-BET-762	BRD2, BRD3, BRD4
	18	OTX015	BRD2, BRD3, BRD4
	19	PFI-1	BRD2, BRD4
	20	I-BET151	BRD3, BRD2, BRD4
	21	(+)-JQ1	BRD4
	22	CPI-203	BRD4, IL-6, MYC
	23	Bromosporine	CECR2, BRD9, BRD4, BRD2
Catechol-O-methyltransferases	24	Entacapone	Catechol-O-methyltransferase (COMT)
Cyclin-dependent Kinases	25	JNJ-7706621	CDK2, CDK1, Aurora A, Aurora B, CDK3, VEGFR2, CDK6, FGFR2, CDK4, GSK-3β, Tie-2, FGFR1, VEGFR3
DNA Methyltransferases	26	Azacitidine	DNA Methyltransferase
	27	Decitabine	DNA Methyltransferase
	28	RG108	DNA methyltransferase
	29	Zebularine	DNA Methyltransferase, Cytidine deaminase
	30	Procainamide HCl	DNA methyltransferase, Sodium channel
	31	SGI-1027	DNMT1, DNMT3B, DNMT3A
Epidermal Growth Factor Receptor	32	AG-490	EGFR
	33	CUDC-101	EGFR, HDAC1, HDAC6, HDAC3, HDAC5, HDAC2, HDAC4, HER2, HDAC10, HDAC9, HDAC8, HDAC7
	34	WHI-P154	EGFR, VEGFR, Src, JAK3
Fms Related Receptor Tyrosine Kinase 3	35	KW-2449	FLT3, Abl, FGFR1, Aurora A, JAK2, Kit, Src
	36	Pacritinib	FLT3, JAK2, TYK2, JAK3
	37	ENMD-2076	FLT3, RET, Aurora A, VEGFR3, Src, NTRK1, CSF-1R, LCK, FAK, PDGFRα, VEGFR2, BLK, FGFR2, YES1, Abl1, FGFR1, Fyn, JAK2, Kit, Aurora B
Histone Acetyltransferases	38	SGC-CBP30	CREBBP, EP300
	39	C646	p300/CBP
Histone Deacetylases	40	AR-42	HDAC
	41	Belinostat	HDAC
	42	Dacinostat	HDAC
	43	M344	HDAC
	44	Panobinostat	HDAC
	45	Scriptaid	HDAC
	46	Sodium Phenylbutyrate	HDAC
	47	Vorinostat	HDAC
	48	Givinostat	HDAC (Class I, IIA, IIB)
	49	Trichostatin A	HDAC (Class I, IIA, IIB)
	50	MC1568	HDAC (Class IIA)
	51	Valproic Acid	HDAC, Autophagy, GABA Receptor
	52	Romidepsin	HDAC1, HDAC2
	53	Quisinostat	HDAC1, HDAC2, HDAC11, HDAC10, HDAC4, HDAC5, HDAC8, HDAC3
	54	Mocetinostat	HDAC1, HDAC2, HDAC11, HDAC3
	55	Tacedinaline	HDAC1, HDAC2, HDAC3
	56	Entinostat	HDAC1, HDAC3

	57	CUDC-907	HDAC1, HDAC3, HDAC10, HDAC2, HDAC11, PI3Kα, HDAC6, PI3Kδ, PI3Kβ
	58	Resminostat	HDAC1, HDAC3, HDAC6
	59	Abexinostat	HDAC1, HDAC3, HDAC6, HDAC2, HDAC10, HDAC8
	60	Pracinostat	HDAC10, HDAC3, HDAC5, HDAC1, HDAC4, HDAC9, HDAC11, HDAC2, HDAC7, HDAC8
	61	RGFP966	HDAC3
	62	RG2833	HDAC3, HDAC1
	63	Nexturastat A	HDAC6
	64	Rocilinostat	HDAC6
	65	Tubacin	HDAC6
	66	Tubastatin A	HDAC6
	67	Tubastatin A HCl	HDAC6
	68	PCI-34051	HDAC8
	69	Droxinostat	HDAC8, HDAC6, HDAC3
	70	TMP269	HDAC9, HDAC7, HDAC5, HDAC4
Histone Demethylases	71	OG-L002	KDM1A
	72	IOX1	KDM3A, KDM4C, KDM6B, KDM2A, KDM4E, KDM5C, PHD2
	73	GSK J4 HCl	KDM6B
Histone Methyltransferases	74	BIX 01294	KMT1C
	75	MM-102	KMT2A
	76	EPZ004777	KMT4
	77	EPZ5676	KMT4
	78	SGC 0946	KMT4
	79	EPZ-6438	KMT6
	80	3-Deazaneplanocin A	KMT6, S-adenosylhomocysteine hydrolase
Hypoxia-inducible Factors	81	IOX2	HIF-1α prolyl hydroxylase-2
	82	2-Methoxyestradiol	HIF-2α, Microtubules depolymerisation, HIF-1α
	83	FG-4592	HIF-α prolyl hydroxylase
Janus Kinases	84	CYT387	JAK1, JAK2, JAK3
	85	Filgotinib	JAK1, JAK2, TYK2, JAK3
	86	AZ 960	JAK2
	87	AZD1480	JAK2
	88	CEP-33779	JAK2
	89	LY2784544	JAK2, FLT3, JAK1, FLT4, FGFR2, TYK2, JAK3, TrkB, FGFR3, KDR, ALK, MUSK, Aurora A, MAP3K9
	90	TG101348	JAK2, FLT3, RET
	91	Ruxolitinib	JAK2, JAK1
	92	S-Ruxolitinib	JAK2, JAK1, TYK2
	93	Baricitinib	JAK2, JAK1, TYK2, JAK3
	94	XL019	JAK2, PDGFRβ, JAK1, FLT3, JAK3
	95	TG101209	JAK2, RET, FLT3, JAK3
	96	WP1066	JAK2, STAT3
	97	NVP-BSK805 2HCl	JAK2, TYK2, JAK3, JAK1
	98	TAK-901	JAK3, c-Src, CLK2, FGR, YES1, LRRK2, FLT3, Fyn, ARG, Axl, Hck, SNF1LK2, Abl, RET, TrkA, LCK, PTK5, Fms, FGFR2, EphB1, EphA1, ARK5, ITK, ALK2, CDK7, BLK, Aurora B, JAK2, EphB2, Aurora A, STK16, EphA2, BRK, EphB4, TNK2, FGFR1, EphA4, STK33, CLK1, AMPK, FES, SLK, Chk2, TYK2, BTK, FAK2, c-Kit, VEGFR2, FGFR3, ALK5, MAP3K9, CLK3, JAK1
	99	AT9283	JAK3, JAK2, Aurora A, Aurora B, Abl1, GSK-3β, FGFR2, VEGFR3, Mer, RET, RSK2, RSK3, TYK2, YES, Abl, DRAK1, FGFR1, FGFR2, FGFR3, VEGFR1, FLT3, PDGFRα, PDK-1, PKCμ, RSK4, Src, VEGFR2
	100	Tofacitinib	JAK3, JAK2, JAK1
	101	Tofacitinib Citrate	JAK3, JAK2, JAK1
	102	ZM 39923 HCl	TGM2, JAK3, EGFR, JAK1
Lethal(3)Malignant Brain Tumor-Like Proteins	103	UNC669	L3MBTL1, L3MBTL3, L3MBTL4
	104	UNC1215	L3MBTL3
Monoamine Oxidases	105	Tranylcypromine HCl	MAO-B, MAO-A, LSD1
O6-Alkylguanine DNA Alkyltransferases	106	Lomeguatrib	MGMT
Poly(ADP-Ribose) Polymerases	107	3-Aminobenzamide	PARP
	108	AZD2461	PARP
	109	INO-1001	PARP
	110	PJ34	PARP
	111	PJ34 HCl	PARP
	112	Rucaparib	PARP
	113	AG-14361	PARP1

	114	Iniparib	PARP1
	115	BMN 673	PARP1, PARP2
	116	Olaparib	PARP2, PARP1
	117	UPF 1069	PARP2, PARP1
	118	Veliparib	PARP2, PARP1
	119	ME0328	PARP3, PARP1
Pim Kinases	120	SMI-4a	Pim1
	121	SGI-1776 free base	Pim1, FLT3, Pim3, Pim2
	122	AZD1208	Pim1, Pim3, Pim2
	123	CX-6258 HCl	Pim1, Pim3, Pim2
Sirtuins	124	Selisistat	SIRT1
	125	SRT1720	SIRT1
	126	Resveratrol	SIRT1, SIRT2, Quinonen reductase 2, IKK β, COX1, COX2, DNA poymerase α, LOX
	127	Quercetin	SIRT1, Src, PKC, PI3Kγ, PI3Kδ, PI3Kβ
	128	Sirtinol	SIRT2, SIRT1

The specific targets of the compounds are listed in order of increasing IC50 values determined in cell-free kinase activity assays (Selleckchem website).

Table S4. Detailed list of all the compounds included in the custom designed compound library for the HDAC inhibitor combination drug screen.

Compound Class	Product Name	Target	Solvent	Concentrations	Company
Apoptosis	YM155	Survivin	DMSO	0.1–1000 nM	Selleckchem
Bcl-2 Family Members	ABT-737	Bcl-2 / Bcl-xL / Bcl-w	DMSO	1–10,000 nM	Selleckchem
	AT101	Bcl-2 / Bcl-xL / Mcl-1	DMSO	1–10,000 nM	Selleckchem
	S63845	Mcl-1	DMSO	1–10,000 nM	Selleckchem
	Venetoclax	Bcl-2	DMSO	1–10,000 nM	Selleckchem
	WEHI-539	Bcl-xL	DMSO	1–10,000 nM	APExBIO
Cell Cycle Regulators	Rabusertib	CHK1	DMSO	1–10,000 nM	Selleckchem
	MK-5108	Aurora Kinase A	DMSO	1–10,000 nM	Selleckchem
	Volasertib	PLK1	DMSO	0.1–1000 nM	Selleckchem
Chemotherapy	Aclarubicin	Topoisomerase I & II	DMSO	0.1–1000 nM	APExBIO
	Cisplatin	DNA Replication	PBS	1–10,000 nM	LUMC Pharmacy
	Doxorubicin	Topoisomerase II	PBS	0.1–1000 nM	LUMC Pharmacy
	Temozolomide	DNA Replication	DMSO	0.01–100 µM	Selleckchem
DNA Damage Repair	Talazoparib	PARP	DMSO	1–10,000 nM	Selleckchem
Metabolism	CB-839	Glutaminase	DMSO	0.01–100 µM	Selleckchem
	Chloroquine Diphosphate	Autophagy / Glutamate Dehydrogenase	PBS	1–100 µM	Selleckchem
	FK866	NAMPT	DMSO	0.008–80 nM	Sigma-Aldrich
	Metformin HCl	AMPK / Glutaminase	RPMI	0.1–10 mM	Selleckchem
	Sapanisertib	mTOR	DMSO	0.01–100 nM	Selleckchem
Protein Degradation	Bortezomib	20S Proteasome	DMSO	0.1–1000 nM	Selleckchem
